# Effects of rapid fluid infusion on hemoglobin concentration: a systematic review and meta-analysis

**DOI:** 10.1186/s13054-022-04191-x

**Published:** 2022-10-23

**Authors:** Armin A. Quispe-Cornejo, Ana L. Alves da Cunha, Hassane Njimi, Wasineenart Mongkolpun, Ana L. Valle-Martins, Mónica Arébalo-López, Jacques Creteur, Jean-Louis Vincent

**Affiliations:** 1 Department of Intensive Care, Erasme University Hospital, Université Libre de Bruxelles, Route de Lennik 808, 1070 Brussels, Belgium; 2 Department of Intensive Care, Hospital da Senhora da Oliveira, Guimarães, Portugal; 3 Department of Intensive Care, Hospital Mater Dei, Belo Horizonte, Brazil; 4 Department of Intensive Care, Hospital Univalle Norte, Cochabamba, Bolivia; 5 Instituto Académico Científico Quispe Cornejo, La Paz, Bolivia

**Keywords:** Hematocrit, Fluid resuscitation, Oxygen delivery, Hemodilution, Fluid challenge

## Abstract

**Background:**

Rapid fluid administration may decrease hemoglobin concentration (Hb) by a diluting effect, which could limit the increase in oxygen delivery (DO_2_) expected with a positive response to fluid challenge in critically ill patients. Our aim was to quantify the decrease in Hb after rapid fluid administration.

**Methods:**

Our protocol was registered in PROSPERO (CRD42020165146). We searched PubMed, the Cochrane Database, and Embase from inception until February 15, 2022. We selected studies that reported Hb before and after rapid fluid administration (bolus fluid given over less than 120 min) with crystalloids and/or colloids in adults. Exclusion criteria were studies that included bleeding patients, or used transfusions or extracorporeal circulation procedures. Studies were divided according to whether they involved non-acutely ill or acutely ill (surgical/trauma, sepsis, circulatory shock or severe hypovolemia, and mixed conditions) subjects. The mean Hb difference and, where reported, the DO_2_ difference before and after fluid administration were extracted. Meta-analyses were conducted to assess differences in Hb before and after rapid fluid administration in all subjects and across subgroups. Random-effect models, meta-regressions and subgroup analyses were performed for meta-analyses. Risk of bias was assessed using the Cochrane Risk of Bias Assessment Tool. Inconsistency among trial results was assessed using the *I*^2^ statistic.

**Results:**

Sixty-five studies met our inclusion criteria (40 in non-acutely ill and 25 in acutely ill subjects), with a total of 2794 participants. Risk of bias was assessed as “low” for randomized controlled trials (RCTs) and ‘low to moderate’ for non-RCTs. Across 63 studies suitable for meta-analysis, the Hb decreased significantly by a mean of 1.33 g/dL [95% CI − 1.45 to − 1.12; *p* < 0.001; *I*^2^ = 96.88] after fluid administration: in non-acutely ill subjects, the mean decrease was 1.56 g/dL [95% CI − 1.69 to − 1.42﻿; *p* < 0.001; *I*^2^ = 96.71] and in acutely ill patients 0.84 g/dL [95% CI − 1.03 to − 0.64; *p* = 0.033; *I*^2^ = 92.91]. The decrease in Hb was less marked in patients with sepsis than in other acutely ill patients. The DO_2_ decreased significantly in fluid non-responders with a significant decrease in Hb.

**Conclusions:**

Hb decreased consistently after rapid fluid administration with moderate certainty of evidence. This effect may limit the positive effects of fluid challenges on DO_2_ and thus on tissue oxygenation.

**Supplementary Information:**

The online version contains supplementary material available at 10.1186/s13054-022-04191-x.

## Introduction

The main objectives of fluid administration in acutely ill patients are to correct hypovolemia and increase cardiac output and oxygen delivery (DO_2_), to restore adequate tissue perfusion [1–3]. However, rapid administration of crystalloids and/or colloids may have a hemodiluting effect, resulting in a decrease in hemoglobin concentration (Hb). As a consequence, even when cardiac output increases, DO_2_ may not increase as much as anticipated. For example, from the DO_2_ equation (DO_2_ = CO × (1.39 × [Hb] × SaO_2_ + (0.003×PaO_2_), at an SaO_2_ of 100%, when Hb decreases from 10 to 9 g/dL cardiac output needs to increase by about 11% to keep DO_2_ steady (and more when the hemoglobin is lower). Additionally, a decrease in Hb after a fluid bolus might be (mis)interpreted as anemia, resulting in an unnecessary blood transfusion [2].

Several studies have reported that fluid administration is associated with a transient decrease in Hb [1–3], which sometimes [3–5], but not always [6, 7], returns rapidly to baseline values after urination. Patients with circulatory shock may show a larger and more persistent hemodiluting effect [1, 2], especially when they are oliguric [8]. However, despite these reports and a clinical perception of Hb reduction after fluid administration, the magnitude of hemodilution in different clinical scenarios has not been objectively assessed.

We therefore conducted a systematic review with meta-analysis to quantify the decrease in Hb after rapid fluid administration in various adult populations.

## Methods

This study was performed according to the PRISMA guidelines and registered on the PROSPERO database on the 28th of April 2020 (CRD42020165146). The protocol is available at: https://www.crd.york.ac.uk/PROSPERO/display_record.php?RecordID=165146.

### Search strategy and selection criteria

We searched PubMed, the Cochrane Database of Systematic Reviews, and Embase from inception until February 15, 2022, to identify all clinical studies in adults (> 18 years) that reported an Hb value before and after rapid fluid administration or fluid challenge with crystalloids and/or colloids of any kind. The full search strings for the three databases, selection strategy, and exclusion criteria are given in Additional file 1. There was no standardized definition of rapid fluid administration, but we excluded studies in which fluid was given over more than 120 min.

The titles and abstracts of the retrieved references were screened independently by three authors (AAQC, ALAC, WM) to assess eligibility for full-text review. The selected full-text articles were then screened independently by the same authors. Any disagreement was resolved by consensus with a fourth author (JLV).

### Data extraction

Information extracted from each study included (full details in Additional file 1):

1. Characteristics of the study.
2. Type of study population: non-acutely ill (healthy volunteers, pre-surgical patients and those with chronic medical conditions) or acutely ill (divided into four subgroups: surgical or trauma, sepsis, circulatory shock and/or severe hypovolemia, and ‘mixed conditions’).
3. Type of interven(tion (type, amount, and duration of fluid administered).
4. Outcome measure (baseline and post-fluid Hb and DO_2_).
5. Information about fluid responsiveness when available, using the definition in the original article.

### Data analysis

The primary analysis was the mean difference with 95% confidence interval (95% CI) in Hb before and after rapid fluid administration (∆Hb). A secondary analysis was the mean difference in DO_2_. Anticipating a high degree of heterogeneity inherent to the differences between different protocols, we used a random-effects model according to the Hartung-Knapp method. We assessed heterogeneity across studies using the *I*^2^ statistic. We analyzed the differences in ∆Hb according to the type of population (non-acutely ill, acutely ill), type of fluid (colloid vs. crystalloid), quantity administered (≤ 250 mL, 250–500 mL, 500–1000 mL, and > 1000 mL during ≤ 1 h; 1000–1500 mL and > 1500 mL during > 1 h), duration of administration (≤ 1 vs. > 1 h), baseline Hb (< 12 g/dL, 12–14 g/dL, > 14 g/dL) and the presence of fluid responsiveness (fluid responder and fluid non-responder). We made no adjustment for multiple comparisons.

For each trial, the risk of bias was evaluated independently by three authors (AAQC, ALAC, WM) using the Cochrane risk of bias tools to evaluate the quality of included randomized controlled trials (RCTs) (Cochrane RoB 2 tool) and non-RCTs (ROBINS-I tool). A fourth author (JLV) resolved any disagreements. Certainty of evidence was assessed by the Grading of Recommendations Assessment, Development and Evaluation (GRADE) tool.

Studies in which the mean ∆Hb with its standard deviation was reported or could be calculated were included in the meta-analysis. If a study presented data in different subgroups of patients each cohort was considered separately for the meta-analysis [9].

The results of studies grouped according to pre-specified study-level characteristics (type of population, subgroups of patients, type of fluid, duration of fluid administration, quantity of fluid administered, different ranges of baseline Hb (8 to 12 g/dL, > 12 to ≤ 14 g/dL, and > 14 g/dL) and presence of fluid responsiveness (fluid responders vs. fluid non-responders as defined in the original studies) were compared using a stratified meta-analysis or random-effects meta-regression.

All analyses were conducted using Stata software, version 17 (StataCorp) with meta command. Statistical significance was considered at the 5% level.

## Results

### Description of included studies

**Fig. 1:**
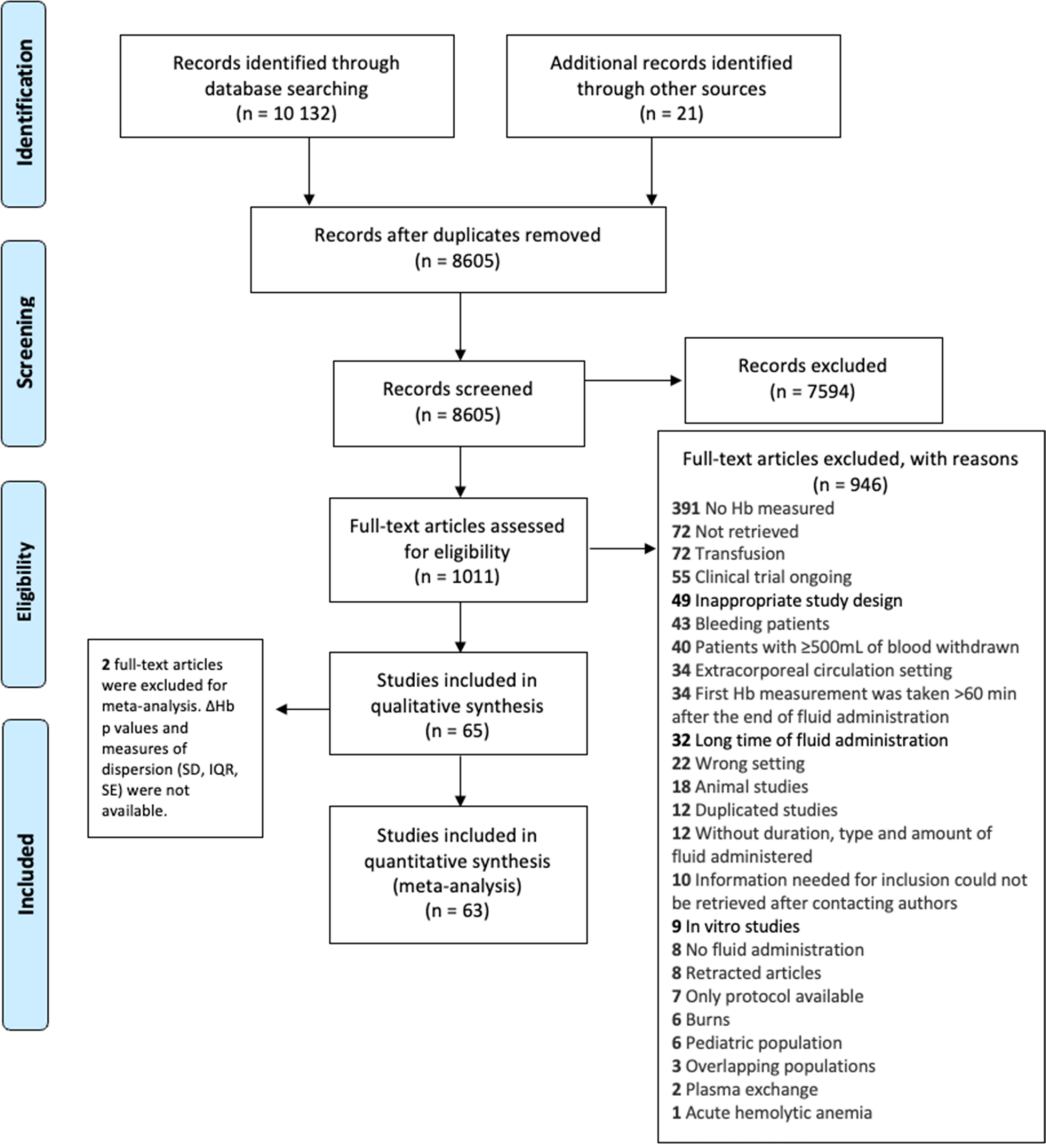
PRISMA flow diagram

The search yielded 8605 studies, 1011 of which underwent full-text screening (Fig. 1). A total of 65 studies, with 157 study sets (subgroups of the included studies) and 2794 subjects met our inclusion criteria for the systematic review; 63 of the 65 studies were eligible for the meta-analysis, with 154 study sets and 2774 subjects. A general visual inspection of the degree of bias detailed in Additional file 1: Table E1 shows ‘low risk’ for RoB 2 and ‘low to moderate risk’ for ROBINS-I [10]. The certainty of evidence was judged as moderate due to minor concerns across (1) imprecision, (2) a considerable amount of heterogeneity, and (3) some risk of publication bias in the acutely ill population.

Forty of the 65 studies included in the systematic review were conducted in non-acutely ill subjects [6, 7, 11–48] and 25 in acutely ill patients, of which 10 were in patients with circulatory shock and/or severe hypovolemia [1, 49–57], 8 were in surgical or trauma patients [58–65], 6 in patients with sepsis [66–71], and 1 in the ‘mixed conditions’ subgroup [72] (Additional file 1: Tables E2 and E3).

For the meta-analysis, 38 of the 63 studies were conducted in non-acutely ill subjects [7, 11–23, 25–27, 29–35, 37–48] and 25 in acutely ill patients, of which 10 were in patients with circulatory shock and/or severe hypovolemia [1, 49, 51–57], 8 in surgical or trauma patients [58–61, 63–65], 6 in patients with sepsis [66–71], and 1 in patients with mixed conditions [72] (Additional file 1: Table E3).

### Primary outcome

**Fig. 2:**
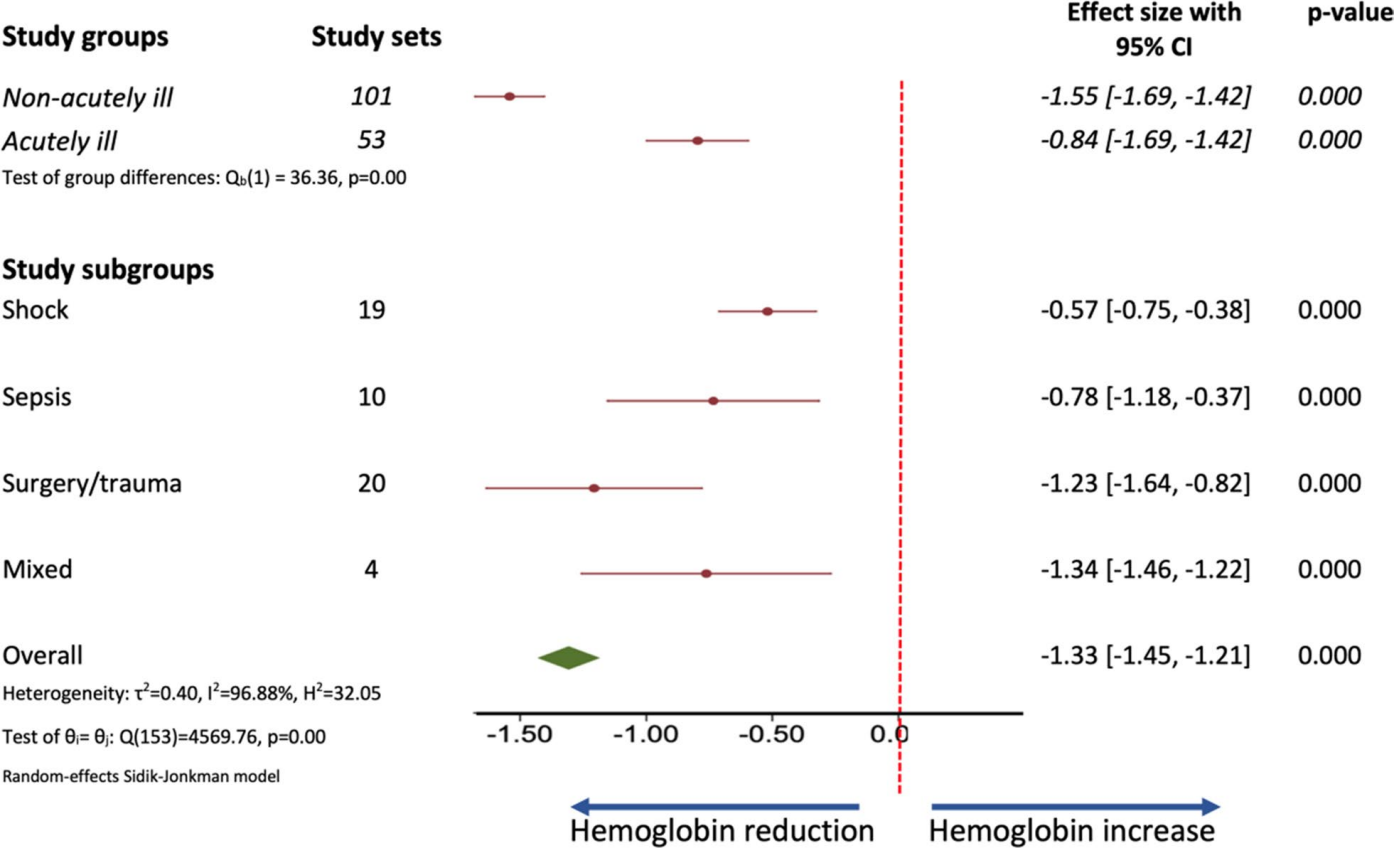
Forest plot of all groups and subgroups

Across all 63 studies included in the meta-analysis, there was a significant decrease in Hb after rapid fluid administration by a mean of 1.33 g/dL [95% CI − 1.45 to − 1.12﻿] (Fig. 2, Additional file 1: Fig. E1).

The decrease in Hb was less marked in the acutely ill (ΔHb − 0.84 g/dL [95% CI − 1.03 to − 0.64]) than in the non-acutely ill (ΔHb − 1.56﻿ g/dL [95% CI − 1.69 to − 1.42﻿]) (*p* < 0.001) (Additional file 1: Table E4, Fig. 2). There was considerable heterogeneity across studies (*I*^2^ = 96.88 overall; 96.71 for the non-acutely ill group and 92.91 for the acutely ill). The effect of small studies was significant for the acutely ill population (*p* < 0.001) but not for the non-acutely ill population (*p* = 0.125).

In the meta-regression analysis, the larger effect size of ∆Hb in the non-acutely ill was associated with longer duration (min) (Additional file 1: Fig. E2) and larger amount (ml) of fluid administration (Additional file 1: Fig. E3).

**Fig. 3:**
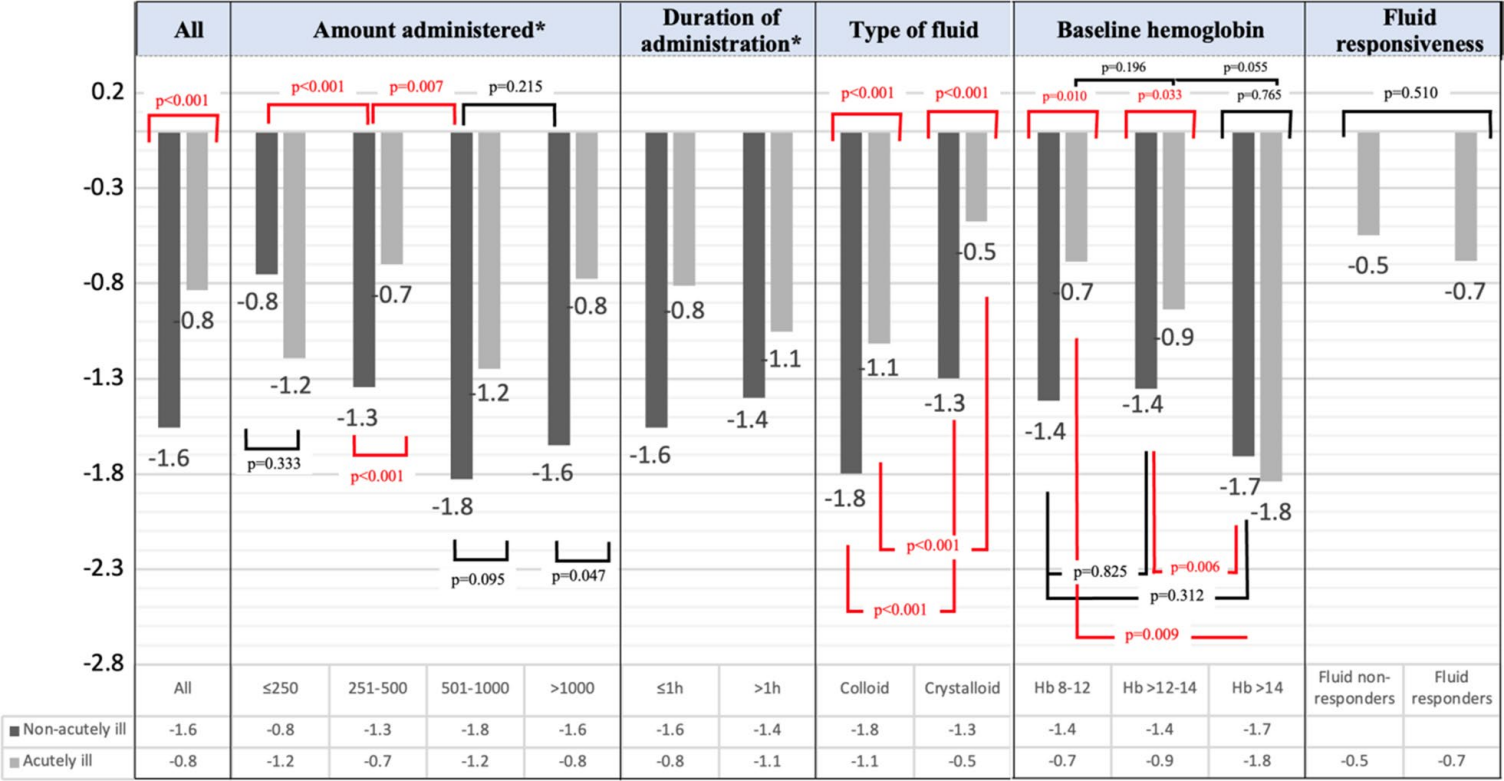
Decrease in hemoglobin concentration (Hb) in non-acutely ill and acutely ill patients according to amount of fluid administered, duration of fluid administration, type of fluid, baseline hemoglobin, and fluid responsiveness

### Subgroup analyses

The results of the subgroup analyses related to change in Hb according to type of fluid, rate of fluid administration, baseline Hb, and fluid responsiveness in the acutely ill population subgroups are shown in Figs. 2, 3, Additional file 1: Figs. E4–E6 and Tables E5–9. The largest decrease in Hb was in the surgery/trauma subgroup (ΔHb − 1.23﻿ g/dL [95% CI − 1.64 to − 0.82]) (Additional file 1: Table E4).

**Fig. 4:**
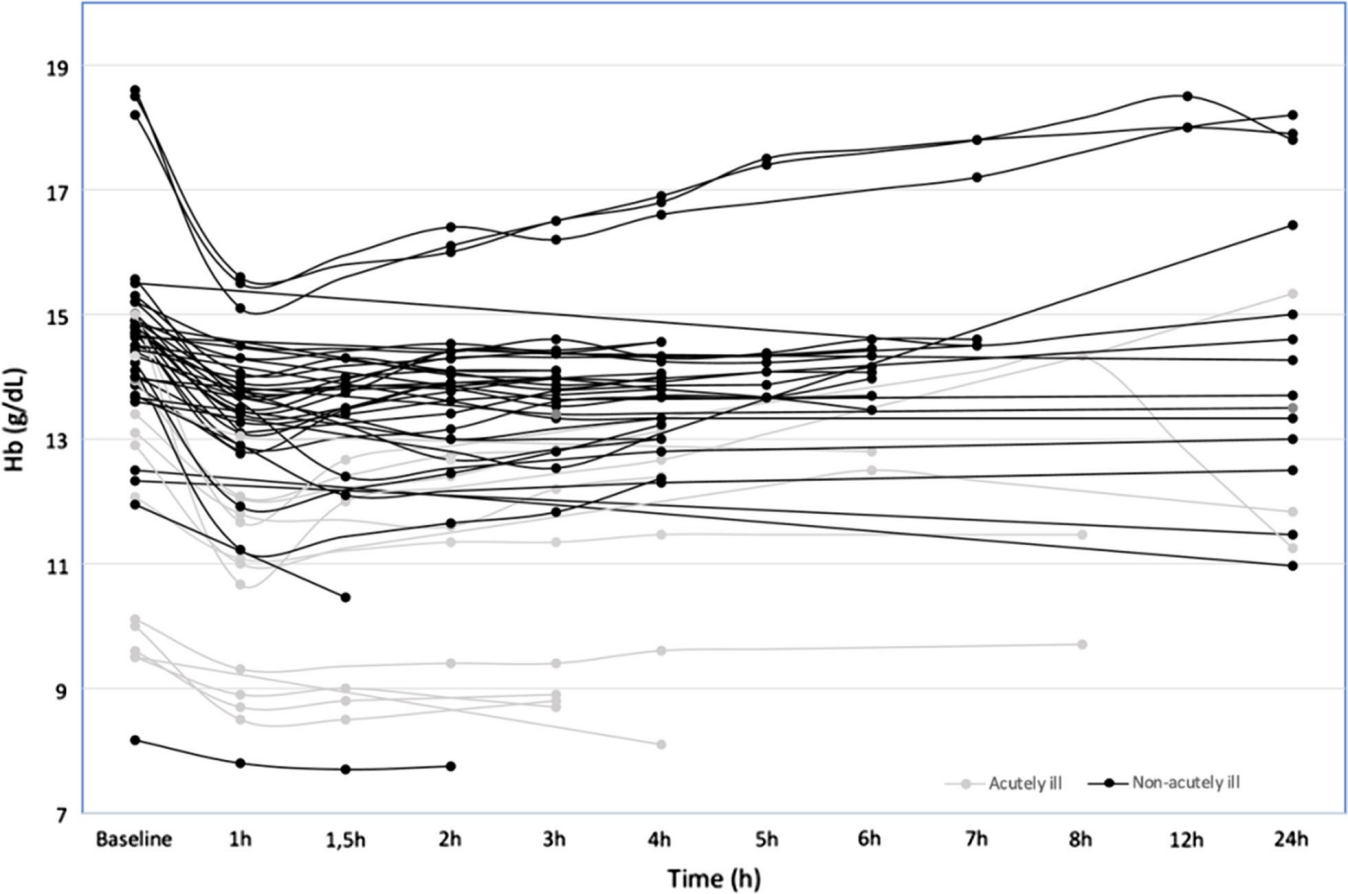
Change in hemoglobin concentration (Hb) from baseline in non-acutely ill (black lines) and acutely ill (gray lines) subjects at different time points

Across all the 157 study sets included in the systematic review, 52 reported more than one measurement after fluid administration. In these patients, the Hb returned to baseline within 8 h in the non-acutely ill and within 5 h in the acutely ill (Fig. 4, Additional file 1: Figs. E6 and E7).

### Fluid type

In the studies included in the meta-analysis, 63 study sets concerned crystalloids, 82 colloids, and 9 a mix of both fluid types (these were not included in the ‘type of fluid’ analysis) (Additional file 1: Table E3). The decrease in Hb was greater with colloids than with crystalloids overall (ΔHb − 1.57 g/dL [95% CI − 1.74 to − 1.40] vs. − 1.07 g/dL [95% CI − 1.21 to − 0.92], *p* < 0.001) and in non-acutely ill and acutely ill groups (Fig. 3 and Additional file 1: Fig. E4 and Table E5). When either crystalloids or colloids were administered, the decrease in Hb in the acutely ill was significantly lower than that in the non-acutely ill (Fig. 3, Additional file 1: Table E5). The decrease in Hb was greater with colloids than with crystalloids in the surgery/trauma subgroup and in patients with mixed conditions (Additional file 1: Table E5 and Fig. E4).

### Duration and amount of fluid

The median duration of infusion was 30 [20–44] minutes in non-acutely ill and acutely ill populations (Additional file 1: Tables E2 and E3). In non-acutely ill subjects, the ΔHb increased with increasing volume of fluid administered over ≤ 1 h, up to 1000 mL (Additional file 1: Table E6 and Figs. E4 and E5); the ΔHb for amounts of fluid > 1000 mL given over > 1 h was − 1.4 g/dL [95%CI − 1.97 to − 0.84] (Additional file 1: Table E7 and Fig. E5). In acutely ill patients, the ΔHb was not directly related to the amount of fluid given (Additional file 1: Tables E6 and E7 and Fig. E5). The decrease in Hb was significantly lower in acutely ill than in non-acutely ill subjects only when an amount of 251–500 mL was administered (Additional file 1: Table E6 and Fig. E5).

### Effect of baseline Hb

The Hb decreased significantly in acutely and non-acutely ill groups regardless of its baseline value but the decrease was greater when the baseline Hb was > 14 g/dL than at lower values (decrease of 17% in acutely ill and 11% in non-acutely ill patients). When the baseline Hb was < 14 g/dL, the decrease in Hb was less marked in acutely ill than in non-acutely ill patients, but similar in acutely ill patients and in non-acutely ill when the baseline Hb was > 14 g/dL (ΔHb − 1.84 g/dL vs. − 1.71 g/dL, *p* = 0.765) (Fig. 3, Additional file 1: Table E8).

### ***Fluid responsiveness and change in DO***_***2***_

Twenty-eight study sets from 15 studies that reported fluid responsiveness were included in the meta-analysis [1, 49, 53–57, 60, 62, 64–67, 69, 71] (Additional file 1: Table E10). All the studies defined fluid responsiveness as an increase in cardiac index of more than 10 or 15% after fluid administration, except one [71] in which fluid responsiveness was defined as a 5% increase in end-tidal carbon dioxide (EtCO2). In every setting analyzed, there was a significant decrease in Hb, which was of a similar magnitude in fluid responders and non-responders (Fig. 3, Additional file 1: Table E9), except in the sepsis subgroup due to a single study that reported an increase in Hb after fluid administration [71]

Twenty-four study sets from 13 studies that reported changes in DO_2_ before and after fluid administration were included in the meta-analysis [1, 49, 54–57, 60, 62, 64–67, 69] (Additional file 1: Table E10). In 11 of the studies, DO_2_ was calculated using the standard formula (DO_2_ = CO × (1.39 × [Hb] × SaO_2_ + (0.003×PaO_2_)); 2 studies [49, 66] did not report how DO_2_ was calculated. There was an overall increase in DO_2_ of 35.8 ml/min/m^2^ [95% CI 13.4–58.2; range − 39 to 120 ml/min/m^2^]. There was a significant difference in the change in DO_2_ between fluid responders (67.76 [95% CI 46.11–89.40]) and non-responders (− 16.30 [95% CI − 31.52 to − 1.09]) (*p* < 0.001) (Additional file 1: Table E11). DO_2_ decreased in the studies in which cardiac index did not increase significantly only when there was a significant decrease in Hb [1, 60] (Additional file 1: Table E12).

## Discussion

Our results show a significant reduction in Hb (a mean of 1.33 g/dL) after fluid administration in all groups of acutely and non-acutely ill subjects, despite marked heterogeneity across studies as evidenced by the high *I*^2^ statistic. This reduction was larger with colloids than with crystalloids. Across the acutely ill population, the largest decrease in Hb was seen in the surgery/trauma subgroup.

Under physiological conditions, a decrease in Hb after rapid fluid administration is both intuitive, through a hemodiluting effect, and counterintuitive, as the kidneys should eliminate the excessive fluid and there may be a fluid shift toward the extravascular space. Interest in this concept was initially raised with the observations by Greenfield and colleagues [73] that rapid crystalloid administration in healthy volunteers was followed by a transient decrease in Hb that started to return toward baseline after 20 min. Studies examining fluid resuscitation in healthy volunteers are, however, different from those in more complex critically ill patients, in whom various dynamic factors act to influence physiological behavior.

The effects of fluid administration vary according to a number of factors, including type and amount of fluid [74–76], renal clearance [14, 23, 24, 77, 78], endothelial integrity [79–82], electrolyte cotransporters, metabolic channels, and aspects associated with a relative shift toward the extravascular space [4]. Colloids are at least one and a half times more effective at volume expansion than crystalloids [83]. As they are expected to remain longer in the bloodstream than crystalloids [84], they may potentially induce a greater reduction in Hb [28], as we observed. Meyer et al. reported that 6% HES (130/0.4) induced sustained hemodilution in critically ill patients with or without sepsis [51]. In patients with sepsis, the decrease in Hb was similar with colloids and crystalloids, suggesting that some capillary leakage may have abolished the differences between the two types of fluids. Moreover, sepsis and circulatory shock are characterized by diffuse endothelial injury and capillary hyperpermeability [14, 23, 79, 81, 82, 85], resulting in greater extravasation of fluid. Studies have shown that 5% or less of a crystalloid infusion remains in the intravascular volume after 1 h in septic patients [80, 86]. In our review, the reduction in Hb was less pronounced in patients with sepsis and shock than in surgery/trauma patients, compatible with greater egress of fluids outside the vascular space in these patients [51, 53].

The amount of fluid given is an important factor in the likely degree of hemodilution induced. The degree by which the Hb decreased was directly related to the amount of fluid given, up to 1000 mL, during the first hour in the non-acutely ill population. However, this trend was not evident in acutely ill patients, likely because of the altered physiological mechanisms in acute illness described previously, notably the fluid extravasation.

In acutely and non-acutely ill populations, the Hb decrease was greatest when the baseline Hb was > 14 g/dL; this may have been due to initial hemoconcentration in some patients [70]. In some groups, the decrease in Hb was as large as 20% (surgery/trauma subgroup) when the baseline Hb was > 14 g/dL. In septic patients, the decrease was more limited, consistent with the expected extravasation phenomenon in patients with sepsis.

In the surgery/trauma subgroup, the type and duration of the procedure [22, 87], the influence of anesthesia on different factors, such as vasodilation and increase in vascular compliance, and a potential reduction in glomerular filtration rate may influence the effect of fluid administration on Hb [88], but we have no data on these aspects. The greater reduction in Hb in the surgical subgroup in our analysis may be explained by the stable and previously healthy condition of many of the patients (elective surgery, interventions performed during induction of anesthesia), physiologically similar to that of non-acutely ill subjects [2, 89].

In the presence of hypovolemia, fluid administration may result in an increase in cardiac output via the Frank-Starling mechanism [90], although this effect may be transient [7, 53], whereas the reduction in Hb may last up to 72 h [91]. This persistent reduction in Hb may limit the ability of fluid administration to achieve the desired objective, i.e., to increase DO_2_ and tissue oxygenation. In subjects in whom Hb decreased, DO_2_ either increased [1, 54–56, 60, 64, 65] or remained stable [62, 66] when cardiac index increased, but decreased [1, 60] when cardiac index did not increase, suggesting that it may be deleterious to administer fluids if cardiac index does not increase.

Of note, hemodilution may also have beneficial effects. For example, hemodilution has been shown to promote cerebral blood flow in preclinical cardiac arrest studies [92, 93]. The decrease in blood viscosity associated with hemodilution can increase red blood cell velocity, facilitating red blood cell influx into the capillaries and therefore improving oxygen transfer to the tissues [94, 95].

## Limitations and strengths

Strengths of this study are the exhaustive review of clinical studies in different settings. We excluded major confounders, such as acute bleeding and transfusion, and classified the risk of bias [96–98].

However, we acknowledge that there was considerable heterogeneity in the included patient populations, in terms of the underlying fluid status of the patients, the fluid tonicity, the timing of the fluid bolus in relation to resuscitation status, the type, amount, and duration of fluid administration, and the timing of observations, which might create bias and limit the interpretation and application of any aggregate quantification. Moreover, there may have been some overlap among groups; for example, some of the patients in the sepsis studies may have had septic shock. Despite our exclusion criteria, in some cases (especially in surgical settings) unrecognized bleeding may have influenced the results. We were also unable to investigate the longer-term effects of rapid fluid administration. None of the studies was specifically designed to evaluate hemoglobin decrease, so we were unable to assess patient-centered outcomes. We extracted some data from graphs, which may have reduced the accuracy of these values, although the participation of two reviewers in this process reduced any observation bias. When needed, we assigned a standard weight and height to calculate fluid amounts and indexes. Finally, the clinical implications of the magnitude of pooled decrease in hemoglobin are not clear, as our study was not designed for this purpose.

## Conclusion

Notwithstanding the study heterogeneity and moderate certainty of evidence, our observations were consistent across studies, showing a systematic decrease in Hb after rapid fluid administration and raising uncertainty about the effects of fluid on DO_2_ in fluid non-responders, i.e., when cardiac index does not increase during the fluid challenge. These observations add a cautious note to the enthusiastic performance of fluid challenges and remind us how important it is to stop the test when cardiac output does not increase.

## Supplementary Information

** Additional file 1.** Online supplementary data.

## Data Availability

Data are available from the corresponding author on reasonable request.

## Abbreviations

CO: Cardiac output
DO_2_: Oxygen delivery
ETCO_2_: End-tidal carbon dioxide
Hb: Hemoglobin
RCT: Randomized controlled trial
