# Supplementary material for: Effects of rapid fluid infusion on hemoglobin concentration: a systematic review and meta-analysis
Source: Crit Care. 2022 Oct 23;26:324. doi: 10.1186/s13054-022-04191-x (PMC9588219; doi:10.1186/s13054-022-04191-x)
Supplement: Supplementary file 1 — Additional file 1. Online supplementary data. [file 13054_2022_4191_MOESM1_ESM.docx]

**Effects of rapid fluid infusion on hemoglobin concentration:**

**A systematic review and meta-analysis**

Quispe-Cornejo AA et al

**Additional File 1**

[Search Strings 2](#_Toc107967676)

[Search strategy and selection criteria 2](#_Toc107967677)

[Data extraction 2](#_Toc107967678)

[Table E1. Risk of bias 2 (RoB 2, on the left) and risk of bias in non-randomized studies – of interventions (ROBINS-I, on the right) 4](#_Toc107967679)

[Table E2. Studies included in the systematic review according to type of population 5](#_Toc107967680)

[Table E3. Description of studies and study sets by group and subgroup for Hb decrease and DO_2_ 11](#_Toc107967681)

[Figure E1. Complete Forest plot with all the included studies 13](#_Toc107967682)

[Table E4. Change in hemoglobin concentration (Hb) after rapid fluid administration in the different study groups 14](#_Toc107967683)

[Figure E2. Metaregression for fluid duration 15](#_Toc107967684)

[Figure E3. Metaregression for fluid amount 15](#_Toc107967685)

[Figure E4. Change in hemoglobin concentration (Hb) from baseline in the four subgroups of acutely ill patients (circulatory shock/severe hypovolemia, sepsis, surgery/trauma, and mixed conditions) according to rate of fluid administration, type of fluid, baseline hemoglobin and fluid responsiveness. 16](#_Toc107967686)

[Figure E5. Decrease in hemoglobin concentration (Hb) in non-acutely ill and acutely ill patients according to rate of fluid administration 17](#_Toc107967687)

[Figure E6. Change in hemoglobin concentration (Hb) from baseline in acutely ill patients at different time points 17](#_Toc107967688)

[Table E5. Delta hemoglobin (Hb) according to type of fluid 18](#_Toc107967689)

[Table E6. Delta hemoglobin (Hb) by ‘amount of fluid (mL) over ≤1h’ 19](#_Toc107967690)

[Table E7. Delta Hb by ‘amount of fluid (mL) over >1h’ 20](#_Toc107967691)

[Table E8. Delta hemoglobin (Hb) for different baseline Hb categories 20](#_Toc107967692)

[Table E9. Delta hemoglobin (Hb) in fluid responders (FR) and non-responders (FNR) 22](#_Toc107967693)

[Figure E7. Change in hemoglobin concentration (Hb) from baseline in non-acutely ill subjects at different time points 22](#_Toc107967694)

[Table E10. Clinical studies that reported fluid responsiveness as defined by change in cardiac index (CI) 23](#_Toc107967695)

[Table E11. Delta oxygen delivery (DO_2_) in fluid responders (FR) and non-responders (FNR) 24](#_Toc107967696)

[Table E12. Changes in oxygen delivery (DO_2_) according to change in cardiac index (CI) in studies showing statistically significant (left) and non-significant (right) decrease in hemoglobin (Hb) 25](#_Toc107967697)

[PRISMA checklist 26](#_Toc107967698)

[References 29](#_Toc107967699)

# Search Strings

***String for PubMed***

((Fluid resuscitation[Title/Abstract] OR fluid bolus[Title/Abstract] OR fluid administration[Title/Abstract] OR fluid challenge[Title/Abstract] OR fluid responsiveness[Title/Abstract] OR hemodilution[Title/Abstract] OR crystalloid[Title/Abstract] OR colloid[Title/Abstract] OR intravenous fluids[Title/Abstract]) AND (hemoglobin OR hematocrit OR blood)).

Filters applied:

Classical Article, Clinical Study, Comparative Study, Controlled Clinical Trial, Journal Article, Meta-Analysis, Multicenter Study, Observational Study, Pragmatic Clinical Trial, Randomized Controlled Trial, Systematic Reviews, Validation Study, Humans, Adult: 19+ years.

***String for Cochrane Database of Systematic Reviews***

-- Title abstract keyword --

(Fluid resuscitation OR fluid bolus OR fluid administration OR fluid challenge OR fluid responsiveness OR hemodilution OR crystalloid OR colloid OR intravenous fluids)

AND

-- All text --

(hemoglobin OR hematocrit OR blood)

***String for EMBASE***

*("Fluid resuscitation" OR "fluid bolus" OR "fluid administration" OR "fluid challenge" OR "fluid responsiveness" OR hemodilution OR crystalloid OR colloid OR "intravenous fluids") in Title****AND***

*("Fluid resuscitation" OR "fluid bolus" OR "fluid administration" OR "fluid challenge" OR "fluid responsiveness" OR hemodilution OR crystalloid OR colloid OR "intravenous fluids") in Abstract****AND***

*("Fluid resuscitation" OR "fluid bolus" OR "fluid administration" OR "fluid challenge" OR "fluid responsiveness" OR hemodilution OR crystalloid OR colloid OR "intravenous fluids") in Keyword Heading Word****AND***

*(hemoglobin OR hematocrit OR blood) in All Fields*

# Search strategy and selection criteria

There was no restriction by language. Animal and *in vitro* studies were not considered. There was no standardized definition of rapid fluid administration, but we excluded studies in which fluid was given over more than 120 min. Only full text publications were considered. References of included papers and related review articles were checked to identify further potentially eligible studies.

*Exclusion criteria*

- no details of the duration, type or amount of fluid administered
- included patients in whom >500 mL of blood was withdrawn for phlebotomy
- the post-fluid administration Hb measurement was taken 60 min or more after the fluid administration
- performed in bleeding patients, in recently transfused patients, in burn patients, in patients receiving extracorporeal circulation, or in patients with acute hemolytic anemia

Titles and abstracts of the retrieved references were screened independently by three authors (AAQC, ALAC, WM) to assess eligibility for full-text review. The selected full-text articles were then screened independently by the same authors. Any disagreement was resolved by a fourth author (JLV).

**Data extraction**

We pilot-tested a data extraction sheet on the first eight included studies and refined it accordingly. Three authors (AAQC, ALAC, WM) extracted the data to this pre-defined table; disagreements were resolved by discussion between two of the authors (AAQC, ALAC). When data were only available from graphics in the original articles, two authors (AAQC, ALAC) discussed and agreed on their interpretation. If several studies reported data from the same patients, we searched across all the studies to extract the required data. Corresponding authors of the individual studies were contacted if possible, when further information was required.

Information extracted from each study included:

1. Characteristics of the study (first author, year, number of patients, and study design).
2. Type of study population: non-acutely ill (healthy volunteers, pre-surgical patients and those with chronic medical conditions) or acutely ill (divided into four subgroups: surgical or trauma, sepsis, circulatory shock and/or severe hypovolemia, and ‘mixed conditions’).
3. Type of intervention (type, amount, and duration of fluid administered). When the amount of fluid was reported in mL/Kg, we calculated the absolute amount using the mean weight reported; when the mean weight was not reported, we assumed a weight of 75 Kg.
4. Outcome measure (baseline and post-fluid Hb and DO_2_). In articles that reported several Hb measurements, we used the first recorded value after the fluid administration for the analyses. When only the hematocrit was available, the value was divided by 3 to obtain the Hb. DO_2_ was reported as ml/min/m^2^. If the DO_2_ indexed value was not reported, it was obtained using a predefined weight of 75 Kg and height of 1.75 m (Mosteller method).
5. When available, we recorded information about fluid responsiveness using the definition in the original article. If the cardiac output was reported instead of the cardiac index, the latter was calculated using a predefined weight of 75 Kg and height of 1.75 m (Mosteller method).

# Table E1. Risk of bias 2 (RoB 2, on the left) and risk of bias in non-randomized studies – of interventions (ROBINS-I, on the right)


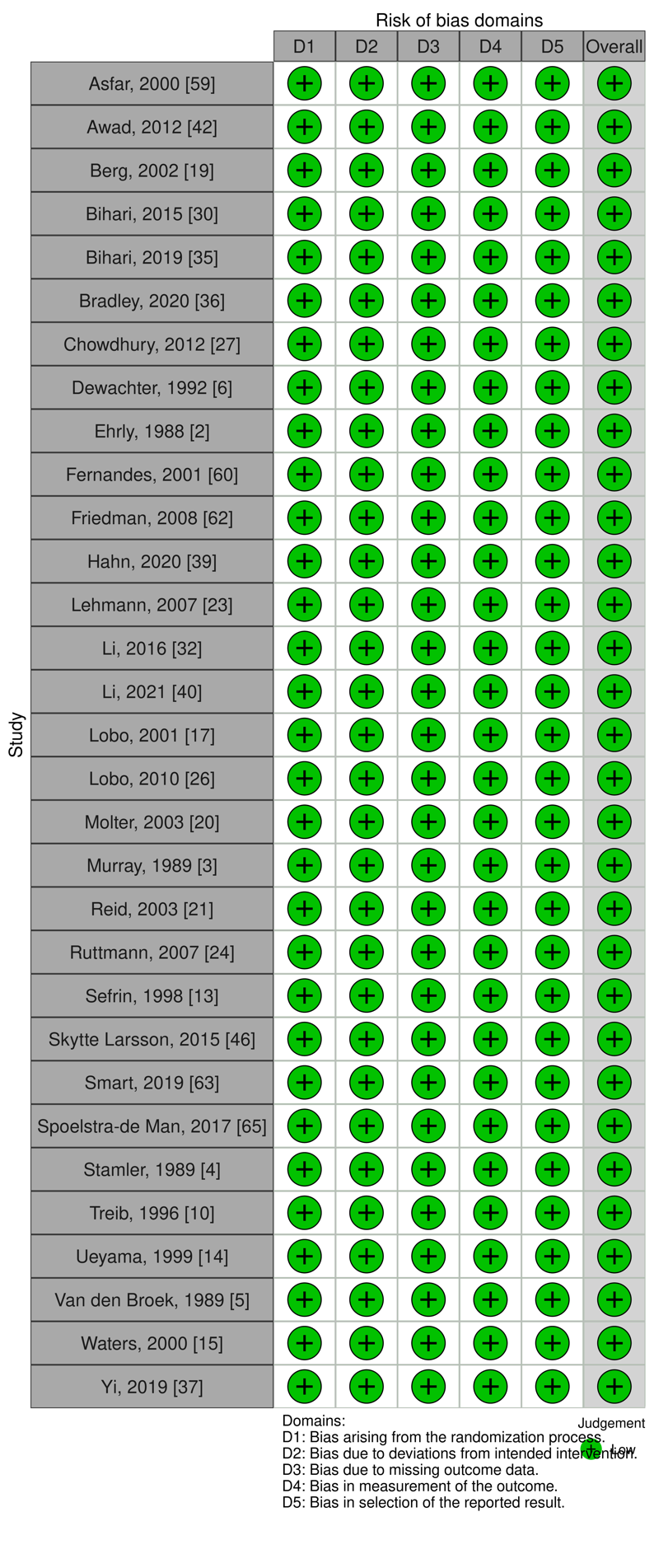

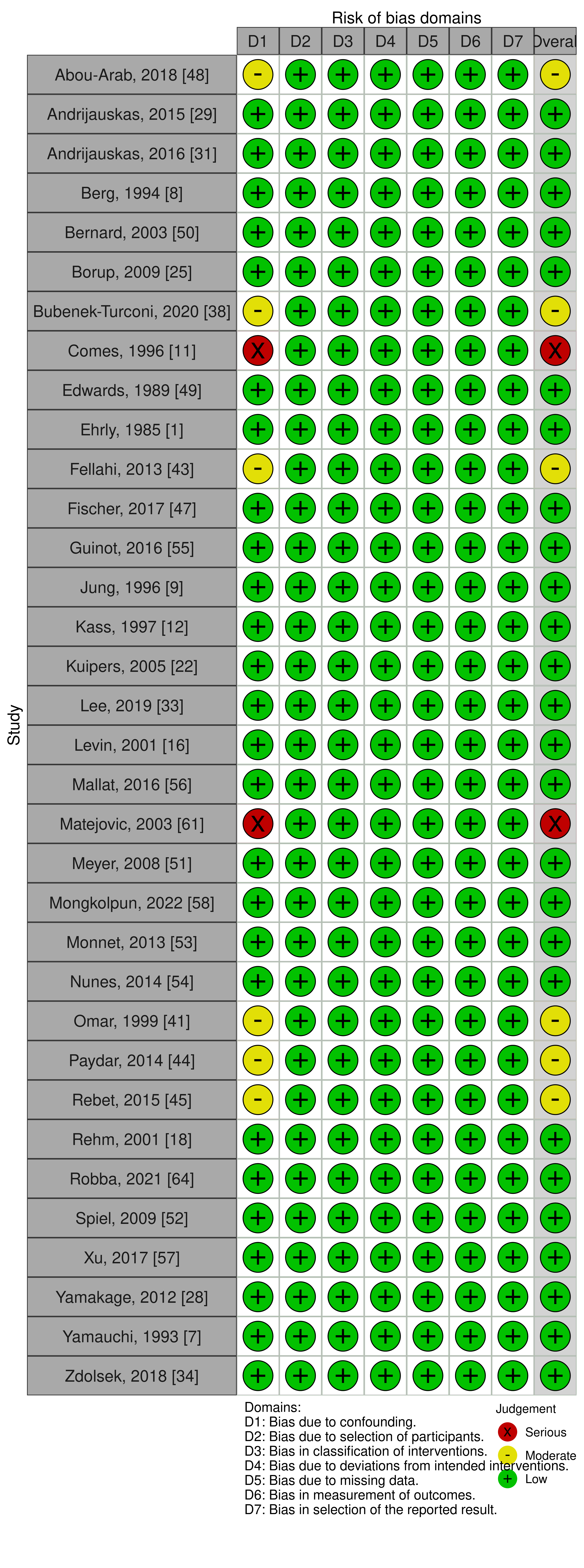


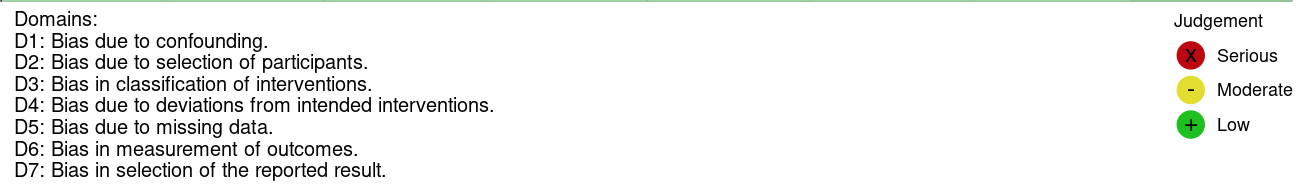

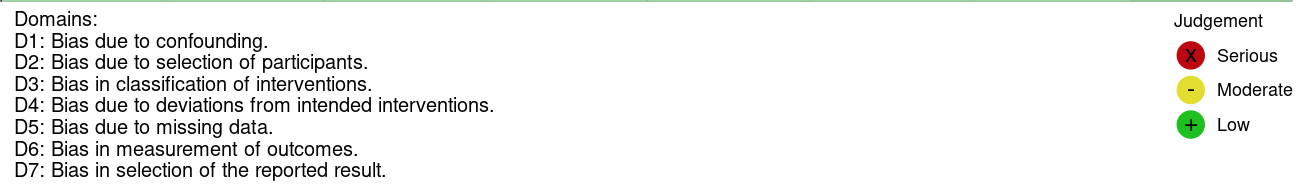

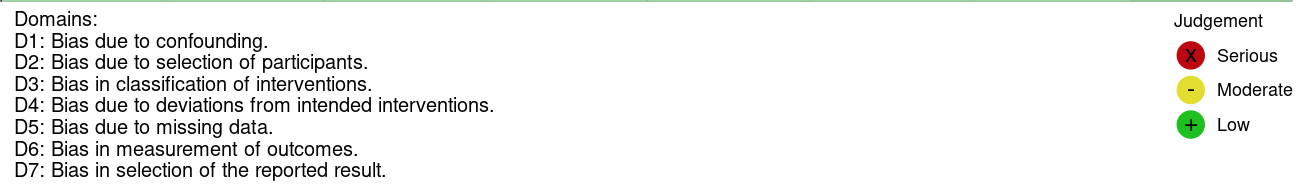

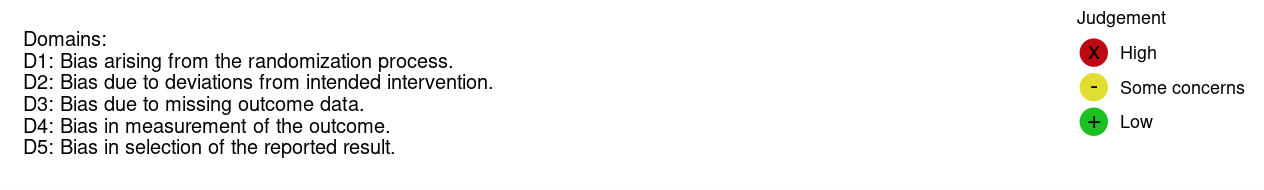


# Table E2. Studies included in the systematic review according to type of population

| **#** | **First author, year (reference)** | **Study design** | **Study population** | **No of sub-**  **jects** | **Fluid type** | | | **Amount of fluid (mL)** | **Amount of fluid (mL/kg)** | **Duration of fluid (min)** | **Baseline Hb**  **(g/dL)** | **Post-fluid Hb**  **(g/dL)** | **∆Hb**  **(g/dl)** | **p value** |
| --- | --- | --- | --- | --- | --- | --- | --- | --- | --- | --- | --- | --- | --- | --- |
|  |  |  |  |  | **Colloid** | **Crystalloid** | |  |  |  |  |  |  |  |
| **NON-ACUTELY ILL POPULATION** | | | | | | | | | | | | | | |
| **1** | Ehrly, 1985 [1] | Interventio-nal | Healthy volunteers | 10 | HES 40000 |  | | 500 |  | 30 | 14.0±1.6 | 13.0±1.5 | -1.0 | 0.001 |
|  |  |  |  | NR | HES 450000^a, b^ |  | | 500 |  | 30 | 14.8 | 14.0 | -0.8 | NR |
| **2** | Ehrly, 1988 [2] | RCT | Intermittent claudication. Interventions were performed 1 week apart. | 10 | HES 6% (HG 200,000/0.62) |  | | 500 |  | 30 | 13.8±1.1^c^ | 12.7±0.9^c^ | -1.1 | <0.005 |
|  |  |  |  | 10 | Dextran 10% 40 |  | | 500 |  | 30 | 14.1±1.0^c^ | 12.3±1.1^c^ | -1.8 | <0.005 |
|  |  |  |  | 10 | HES 10% (HG 200,000/0.62) |  | | 500 |  | 30 | 14.0±1.0^c^ | 12.4±1.1^c^ | -1.6 | <0.005 |
| **3** | Murray, 1989 [3] | RCT | Study done before cesarean | 30 |  | RL | | 2000 |  | 20 | 12.3 ±1.4 | 10.6 ±1.0 | -1.7 | NR |
|  |  |  |  | 30 | HES 6% |  | | 1000 |  | 20 | 12.5±0.8 | 10.2 ±1.1 | -2.3 | NR |
| **4** | Stamler, 1989 [4] | RCT | Healthy volunteers | 20 |  | NS, Group 1, 1 bolus | | 1500^d^ | 20 | 45 | 14.3±1.1^c^ | 12.7±1.0^c^ | -1.6 | <0.001 |
|  |  |  |  | 20 |  | NS Group 1, 2 boluses | | 2625^d^ | 20 + 15 | 105 | 14.3±1.1^c^ | 12.9±1.0^c^ | -1.4 | <0.001 |
|  |  |  |  | 20 |  | NS Group 2, 1 bolus | | 1500^d^ | 20 | 45 | 14.8±0.9^c^ | 13.3±1.1^c^ | -1.5 | <0.001 |
| **5** | Van Den Broek, 1989 [5] | RCT | Healthy volunteers undergoing extra-corporeal shock wave lithotripsy | 27 | Gelatin |  | | 750 |  | 15 | 14.0±0.0^c^ | 12.3±0.0^c^ | -1.7 | <0.05 |
|  |  |  |  | 28 |  | Dextrose + NS | | 750 |  | 15 | 14.3±0.0^c^ | 12.0±0.0^c^ | -2.3 | <0.05 |
|  |  |  |  | 27 |  | RL | | 750 |  | 15 | 13.7±0.0^c^ | 13.0±0.0^c^ | -0.7 | ns |
| **6** | Dewachter, 1992 [6] | RCT | Healthy, before dental surgery | 10 | Albumin 4% |  | | 500 |  | 30 | 14.5±1.1^c^ | 13.0±1.4^c^ | -1.5 | ns |
|  |  |  |  | 10 | Dextran 40, 3.5% |  | | 500 |  | 30 | 14.7±1.0^c^ | 12.7±1.4^c^ | -2.0 | ns |
|  |  |  |  | 10 | Dextran 60, 6% |  | | 500 |  | 30 | 14.4±1.8^c^ | 12.1±1.9^c^ | -2.3 | ns |
|  |  |  |  | 10 | Hidroethylalmidon 6% |  | | 500 |  | 30 | 13.6±0.7^c^ | 11.7±1.0^c^ | -1.9 | ns |
|  |  |  |  | 10 | Gelatin modified |  | | 500 |  | 30 | 14.0±1.0^c^ | 12.4±1.2^c^ | -1.5 | ns |
|  |  |  |  | 10 |  | RL | | 500 |  | 30 | 14.6±1.7^c^ | 13.4±1.4^c^ | -1.3 | ns |
| **7** | Yamauchi, 1993 [7] | Interventio-nal | Stroke  Phlebotomy of 400 mL before RFA | 5 | HES 6% |  | | 400 |  | 30-40 | 13.8±1.2 | 12.2±1.1 | -1.6 | <0.05 |
| **8** | Berg, 1994 [8] | Interventio-nal | Healthy medical students | 12 |  | RA | | 1000 |  | 40 | 13.8±1.1 | 13.2±1.1 | -0.6 | <0.01 |
| **9** | Jung, 1996 [9] | Interventio-nal |  | 10 | HES 100/0.5, 10% |  | | 500 |  | 60 | 15.6±1.0^c,e^ | 13.7±1.3^c,e,f^ | -1.9 | 0.0001 |
| **10** | Treib, 1996 [10] | RCT | Cerebrovascular diseases | 6 | HES 6%, 200/0.62 |  | | 500 |  | 52.5 | 14.7 ±0.5^c^ | 13.3±0.8^c^ | -1.4 | NR |
|  |  |  |  | 6 | HES 10%, 200/0.62 |  |  | 500 |  | 52.5 | 14.5 ±0.7^c^ | 12.0±0.6^c^ | -2.5 | NR |
| **11** | Comes, 1996 [11] | Interventio-nal | Acute ischemic stroke | 18 | HES 6% |  | | 250 |  | 60 | 14.8±0.4^c,f^ | 14.2±0.1^c,f^ | -0.6 | <0.05 |
| **12** | Kass, 1997 [12] | Interventio-nal | Healthy volunteers | 20 |  | NS | | 1146^g^ | 15 | 30 | 13.8±0.9^c^ | 12.3±0.8^c^ | -1.5 | <0.01 |
| **13** | Sefrin, 1998 [13] | RCT | Elective vertebral disc surgery | 9 | HES potato |  | | 1000 |  | 30 | 14.8±1.1^f^ | 12.5±1.2^f^ | -2.3 | NR |
|  |  |  |  | 11 | HES corn |  | | 1000 |  | 30 | 13.7±1.4^f^ | 11.9±1.7^f^ | -1.8 | NR |
| **14** | Ueyama, 1999 [14] | RCT | Healthy, before elective Caesarean section | 12 |  | RL | | 1500 |  | 30 | 10.9±0.7 | 9.8±0.8 | -1.1 | <0.01 |
|  |  |  |  | 12 | HES 6%, 0.5L |  | | 500 |  | 30 | 11.0±0.8 | 9.9±1.0 | -1.1 | <0.01 |
|  |  |  |  | 12 | HES 6%, 1L |  | | 1000 |  | 30 | 11.0±0.9 | 8.9±0.7 | -2.1 | <0.01 |
| **15** | Waters, 2000 [15] | RCT | Healthy volunteers | 10 | HES 6% |  | | 1095 | 15 | 30 | 15.3±1.3^c^ | 13.1±1.1^c^ | -2.2 | NR |
|  |  |  |  | 10 | Albumin 5% |  | | 1094 |  | 30 | 15.2 ±1.2^c^ | 13.7±1.2^c^ | -1.5 | NR |
| **16** | Levin, 2001 [16] | Interventio-nal | Healthy male volunteers | 9 |  | NS | | 1000 |  | “rapid” | 14.9 ±1.2^c^ | 14.0±0.8^c^ | -0.9 | 0.03 |
| **17** | Lobo, 2001 [17]^b^ | RCT | Healthy male volunteers | 10 |  | NS | | 2000 |  | 60 | 14.9[13.9-15.9]^h^ | 13.9^i^ | -1.0 | NR |
| **18** | Rehm, 2001 [18] | Interventio-nal | Before major gynecological procedures | 10 | Albumin 5% |  | | 1379 | 20 | 15 | 11.3 ±1.0^c^ | 9.2±0.6^c^ | -2.1 | <0.05 |
|  |  |  |  | 10 | HES 6% |  | | 1417 |  | 15 | 11.8±1.5^c^ | 9.1±1.2^c^ | -2.7 | <0.05 |
| **19** | Berg, 2002 [19] |  |  | 12 | Albumin 40 g/L |  | | 546 | 7.1 | 30 | 14.7±0.2^c,f^ | 13.9±0.3^c,f^ | -0.8 | ns |
|  |  | RCT | Healthy volunteers | 12 |  | RA | | 1637 | 21.4 | 30 | 14.5±0.3^c,f^ | 13.5±0.3^c,f^ | -1.0 | ns |
|  |  |  |  | 12 | Dextran 30 g/L |  | | 546 | 7.1 | 30 | 14.4±0.2 ^c,f^ | 13.7±0.3^c,f^ | -0.7 | ns |
| **20** | Molter, 2003 [20] | RCT | Before major elective cardiac surgery | 10 | HES 10% 200/0.5 + NS | NS | | 1440^j^ |  | 2-5 | 13.8±1.4^c^ | 11.8±1.0^c^ | -2.0 | ns |
|  |  |  |  | 9 | Dextran 10% 40 + NS |  | | 1248^j^ |  | 2-5 | 13.2±1.0^c^ | 11.1±1.0^c^ | -2.1 | ns |
|  |  |  |  | 8 | HS 7.2% + HES 200/0.5 |  | | 1248^j^ |  | 2-5 | 13.7±1.9^c^ | 11.5±1.6^c^ | -2.2 | ns |
|  |  |  |  | 8 | HS 7.2% + Dextran 10% 60 |  | | 288^j^ |  | 2-5 | 14.0±1.1^c^ | 13.0±1.6^c^ | -1.0 | ns |
|  |  |  |  | 8 |  |  | | 288^j^ |  | 2-5 | 13.4±1.5^c^ | 12.3±2.3^c^ | -1.1 | ns |
| **21** | Reid, 2003 [21] | RCT | Healthy male volunteers | 9 |  | NS | | 2000 |  | 60 | 15.2 [14.5-16.0]^h^ | 13.8 (0.9)^e,k^ | -1.4 | NR |
|  |  |  |  | 9 |  | RL | | 2000 |  | 60 | 15.3 [14.4-15.7]^h^ | 14.1 (0.7)^e,k^ | -1.2 | NR |
| **22** | Kuipers, 2005 [22] | Interventio-nal | Healthy volunteers | 7 |  | NS | | 500 |  | 10-13 | 15.2±0.9 | 14.5±1.0 | -0.7 | <0.01 |
| **23** | Lehmann, 2007 [23] | RCT | Healthy volunteers | 8 | HES 130/0.42/6:1 |  | | 1000 |  | 30 | 18.6±1.4^f^ | 15.3±1.1^f^ | -3.3 | NR |
|  |  |  |  | 8 | HES 130/0.4/9:1 |  | | 1000 |  | 30 | 18.5±1.2^f^ | 15.7±1.0^f^ | -2.8 | NR |
|  |  |  |  | 8 | HES 200/0.5/5:1 |  | | 1000 |  | 30 | 18.2±1.5^f^ | 15.5±0.9^f^ | -2.7 | NR |
| **24** | Ruttmann, 2007 [24] |  |  | 25 |  | NS | | 1050^d^ | 14 | 30 | 13.8±0.7^c^ | 12.4±0,8^c^ | -1.4 | NR |
|  |  | RCT | Healthy volunteers | 25 |  | NS + 1.5 g/L Mg^2+^ | | 1050^d^ |  | 30 | 13.8±0.7^c^ | 12.4±0.8^c^ | -1.4 | NR |
|  |  |  |  | 25 |  | NS + 3 g/L Mg^2+^ | | 1050^d^ |  | 30 | 13.8±0.7^c^ | 12.3±0.8^c^ | -1.5 | NR |
| **25** | Borup, 2009 [25]^b^ | Interventio-nal | Pre-operative elective cholecystectomy | 10 |  | RL | | 951 | 12.5 | 30 | 8.2 [6.8-9.5]^h^ | 7.6^f^ | -0.6 | NR |
| **26** | Lobo, 2010 [26] | RCT | Healthy male volunteers | 10 |  | NS | | 1000 |  | 60 | 14.9±0.3^e^ | 14.3±0.1^e,f^ | -0.6 | NR |
|  |  |  |  | 10 | Gelatin 4% |  | | 1000 |  | 60 | 14.9±0.3^e^ | 13.1±0.1^e,f^ | -1.8 | NR |
|  |  |  |  | 10 | HES 6% |  | | 1000 |  | 60 | 14.7±0.4^e^ | 12.9±0.3^e,f^ | -1.8 | NR |
| **27** | Chowdhury, 2012 [27] | RCT | Healthy volunteers | 12 |  | NS | | 2000 |  | 60 | 14.8±0.2^e^ | 13.4±0.10^f^ | -1.4 | NR |
|  |  |  |  | 12 |  | Isotonic electrolyte solution | | 2000 |  | 60 | 14.8±0.2^e^ | 13.1±0.25^f^ | -1.7 | NR |
| **28** | Yamakage, 2012 [28] | Interventio-nal | Healthy male volunteers | 12 | HES 6%, 130/0.4 |  | | 500 |  | 30 | 15.5±0.4 | 13.8±0.4 | -1.7 | NR |
| **29** | Andrijauskas, 2015 [29] | Interventio-nal | Preoperative | 36 |  | RA, 1 bolus | | 423^l^ | 5 | 4^n^ | 12.7±0.7^n^ | 11.9±0.8^n^ | -0.8 | NR^O^ |
|  |  |  |  | 36 |  | RA, 2 boluses | | 845^l^ | 10 | 13^n^ | 12.7±0.7^n^ | 11.7±0.8^n^ | -1 | NR^O^ |
|  |  |  |  | 36 |  | RA, 3 boluses | | 1268^l^ | 15 | 22^n^ | 12.7±0.7^n^ | 11.6±0.8^n^ | -1.1 | NR^O^ |
| **30** | Bihari, 2015 [30] | RCT | Healthy volunteers | 6 | Albumin 4% |  | | 2400^p^ | 30 | 40 | 15±0.7^c^ | 12±1.0^c^ | -3.0 | 0.000 |
|  |  |  |  | 6 |  | NS | | 2400^p^ |  | 40 | 15±0.7^c^ | 12.7±0.7^c^ | -2.3 | 0.000 |
| **31** | Andrijauskas, 2016 [31] | Interventio-nal | Preoperative | 48 |  | RA, 1 bolus | | 218^q^ | 2.5 | 2.5^r^ | 13.0±1.0^n^ | 12.1±1.0^n^ | -0.9 | NR^O^ |
|  |  |  |  | 48 |  | RA, 2 boluses | | 435^q^ | 5 | 10^r^ | 13.0±1.0^n^ | 11.8±1.0^n^ | -1.2 | NR^O^ |
|  |  |  |  | 48 |  | RA, 3 boluses | | 653^q^ | 7.5 | 17.5^r^ | 13.0±1.0^n^ | 11.7±1.0^n^ | -1.3 | NR^O^ |
|  |  |  |  | 48 |  | RA, 4 boluses | | 870^q^ | 10 | 25^r^ | 13.0±1.0^n^ | 11.6±1.0^n^ | -1.4 | NR^O^ |
|  |  |  |  | 48 |  | RA, 5 boluses | | 1088^q^ | 12.5 | 32.5^r^ | 13.0±1.0^n^ | 11.5±0.9^n^ | -1.5 | NR^O^ |
|  |  |  |  | 48 |  | RA, 6 boluses | | 1305^q^ | 15 | 40^r^ | 13.0±1.0^n^ | 11.4±1.0^n^ | -1.6 | NR^O^ |
| **32** | Li, 2016 [32] | RCT | Pre-operative  group | 20 |  | RL | | 375^d^ | 5 | 50 | 12.7±1.5 | 12.3±1.6 | -0.4 | <0.001 |
|  |  |  |  | 20 | RL+HES 130/0.4, 6% | RL+HES 130/0.4, 6% | | 2250^d^ | 10 RL + 20 HES^s^ | 50 | 13.3 ±1.8 | 10.4 ±1.6 | -2.9 | <0.001 |
| **33** | Lee, 2019 [33] | Interventio-nal | Healthy volunteers | 27 |  | RL | | 1000 |  | 20 | 11.9 [10.8-13.1]^n,h^ | 11.0 [9.5-12.1] ^n,h^ | -0.9 | <0.001^n^ |
| **34** | Zdolsek, 2018 [34] | Interventio-nal | Anesthetized patients prior to open gastrectomy | 15 | Albumin 20% |  | | 231^t^ | 3 | 30 | 13.6±0.8^n^ | 12.5±0.8^n^ | -1.1 | NR^o^ |
| **35** | Bihari, 2019 [35] | RCT | Healthy volunteers | 6 | Albumin 4% |  | | 240 | 30 | 24 | 16.0±1.2 | 13.5±2.4 | -2.5 | 0.017 |
|  |  |  |  | 6 | Albumin 20% |  | | 477 | 6 | 20 | 15.7±1.5 | 13.5±1.3 | -2.2 | 0.000 |
|  |  |  |  | 6 |  | NS | | 2400 | 30 | 24 | 16.0±1.4 | 14.6±1.2 | -1.4 | 0.001 |
|  |  |  |  | 6 |  | RL | | 2400 | 30 | 24 | 16.0±1.7 | 14.9±1.2 | -1.1 | 0.01 |
| **36** | Bradley, 2020 [36] | RCT | Healthy male volunteers | 10 |  | Isotonic electrolyte solution | | 1500 |  | 60 | 15.0±0.2 | 13.5±0.0^f,k^ | -1.5 | NR |
|  |  |  |  | 10 | Gelatin 4% |  | | 500 |  | 60 | 14.8±0.3 | 13.5±0.1^f,k^ | -1.3 | NR |
|  |  |  |  | 10 | Gelatin 4% + isotonic electrolyte solution | Gelatin 4% + isotonic electrolyte solution | | 1500 |  | 60 | 14.7±0.3 | 12.8±0.2^f,k^ | -1.9 | NR |
| **37** | Yi, 2019 [37] | RCT | Healthy male volunteers | 12 | HES 10%, 1 bolus |  | | 100^u^ |  | 6^v^ | 14.1±0.9^n^ | 13.5±0.9^n^ | -0.6 | NR^O^ |
|  |  |  |  | 12 | HES 10%, 3 boluses |  |  | 300^u^ |  | 18^v^ | 14.1±0.9^n^ | 12.8±0.8^n^ | -1.3 |  |
|  |  |  |  | 12 | HES 10%, 5 boluses |  |  | 500^u^ |  | 30^v^ | 14.1±0.9^n^ | 12.1±0.9^n^ | -2.0 |  |
|  |  |  |  | 12 | HES 10%, 7 boluses |  |  | 700^u^ |  | 42^v^ | 14.1±0.9^n^ | 11.8±0.8^n^ | -2.3 |  |
|  |  |  |  | 12 | HES 10%, 9 boluses |  |  | 900^u^ |  | 54^v^ | 14.1±0.9^n^ | 11.3±0.8^n^ | -2.8 |  |
|  |  |  |  | 12 | HES 10%, 10 boluses |  |  | 1000^u^ |  | 60^v^ | 14.1±0.9^n^ | 11.2±0.7^n^ | -2.9 |  |
|  |  |  |  | 12 |  | RL, 1 bolus | | 270^w^ | 40 (RL) | 6^v^ | 13.9±0.6^n^ | 13.1±0.6^n^ | -0.8 |  |
|  |  |  |  | 12 |  | RL, 3 boluses | | 810^w^ |  | 18^v^ | 13.9±0.6^n^ | 12.5±0.6^n^ | -1.4 |  |
|  |  |  |  | 12 |  | RL, 5 boluses | | 1350^w^ |  | 30^v^ | 13.9±0.6^n^ | 12.2±0.5^n^ | -1.7 |  |
|  |  |  |  | 12 |  | RL, 7 boluses | | 1890^w^ |  | 42^v^ | 13.9±0.6^n^ | 12.0±0.5^n^ | -1.9 |  |
|  |  |  |  | 12 |  | RL, 9 boluses | | 2430^w^ |  | 54^v^ | 13.9±0.6^n^ | 11.9±0.5^n^ | -2.0 |  |
|  |  |  |  | 12 |  | RL, 10 boluses | | 2700^w^ |  | 60^v^ | 13.9±0.6^n^ | 12.0±0.5^n^ | -1.9 |  |
|  |  |  |  | 12 | HES 6%,  1 bolus |  | | 100^u^ |  | 6^v^ | 14.2±0.9^n^ | 13.6±0.9^n^ | -0.6 |  |
|  |  |  |  | 12 | HES 6%,  3 boluses |  |  | 300^u^ |  | 18^v^ | 14.2±0.9^n^ | 13.1±0.9^n^ | -1.1 |  |
|  |  |  |  | 12 | HES 6%,  5 boluses |  |  | 500^u^ |  | 30^v^ | 14.2±0.9^n^ | 12.7±0.9^n^ | -1.5 |  |
|  |  |  |  | 12 | HES 6%,  7 boluses |  |  | 700^u^ |  | 42^v^ | 14.2±0.9^n^ | 12.2±0.9^n^ | -2.0 |  |
|  |  |  |  | 12 | HES 6%,  9 boluses |  |  | 900^u^ |  | 54^v^ | 14.2±0.9^n^ | 11.9±1.0^n^ | -2.3 |  |
|  |  |  |  | 12 | HES 6%, 10 boluses |  |  | 1000 |  | 60^v^ | 14.2±0.9^n^ | 11.9±0.8^n^ | -2.3 |  |
| **38** | Bubenek-Turconi, 2020 [38] | Interventio-nal | After induction, before major surgery | 40 | Gelatin 4% |  | | 250 |  | 1 | 11.9±1.7 | 11.3±1.5 | -0.6 | <0.001 |
| **39** | Hahn, 2020 [39] | RCT | Presurgical volunteers (hysterectomy) | 25 |  | RL | | 1869 | 25 | 30 | 12.0±1.6 | 9.6±1.4 | -2.4 | NR |
| **40** | Li, 2021 [40] | RCT | Healthy male volunteers | 14 |  | NS | | 2352^x^ | 30 | 20 | 15.0 | 14.7 | -0.3 | <0.001 |
| **ACUTELY ILL POPULATION** | | | | | | | | | | | | | | |
| **Surgical/trauma patients** | | | | | | | | | | | | | | |
| **1** | Omar, 1999 [41] | Interventio-nal | During prostatectomy | 25 | HES 6%, 200 kD |  | | 200 |  | 20 | 15.0±0.9^c^ | 14.0±0.7^c^ | -1.0 | S |
|  |  |  |  | 25 | HES 6%, 200 kD |  |  | 400 |  | 40 | 15.0±0.9^c^ | 11.7±0.7^c^ | -1.3 | S |
|  |  |  |  | 25 | HES 6%, 200 kD |  |  | 600 |  | 60 | 15.0±0.9^c^ | 10.7±0.5^c^ | -2.3 | S |
|  |  |  |  | 25 | HES 6%, 200 kD |  |  | 900 |  | 90 | 15.0±0.9^c^ | 12±0.6^c^ | -3.0 | S |
|  |  |  |  | 25 | Albumin 5% |  |  | 200 |  | 20 | 15.0±0.8^c^ | 13,3±0.6^c^ | -1.7 | S |
|  |  |  |  | 25 | Albumin 5% |  |  | 400 |  | 40 | 15.0±0.8^c^ | 12±0.5^c^ | -3.0 | S |
|  |  |  |  | 25 | Albumin 5% |  |  | 600 |  | 60 | 15.0±0.8^c^ | 11.7±0.6^c^ | -3.3 | S |
|  |  |  |  | 25 | Albumin 5% |  |  | 900 |  | 90 | 15.0±0.8^c^ | 12.7±0.6^c^ | -2.3 | S |
| **2** | Awad, 2012 [42] | RCT | During elective laparoscopic cholecystectomy | 13 | HES 6%, 130 kDa |  | | 1000 |  | 60 | 13.1±0.5^e^ | 11.8±0.4^f^ | -1.3 | NR |
|  |  |  |  | 12 | Gelatin 4%, 30 kDa |  |  | 1000 |  | 60 | 13.4±0.2^e^ | 12.1±0.4^f^ | -1.3 | NR |
| **3** | Fellahi, 2013 [43] | Prospective observation-nal | After cardiac surgery | 37 | HES 130/0.4 6% (FR) |  | | 500 |  | 15 | 11.8±1.6 | 10.4±1.5 | -1.4 | <0.01 |
|  |  |  |  | 13 | HES 130/0.4 6% (FNR) |  |  | 500 |  | 15 | 11.1±1.4 | 9.9±1.1 | -1.2 | <0.01 |
| **4** | Paydar, 2014 [44] | Interventio-nal | Stable (non-bleeding) polytrauma | 84 |  | NS | | 1000 |  | 60 | 13.7±1.6 | 13.1±1.8 | -0.6 | <0.001 |
| **5** | Rebet, 2015 [45] | Prospective observation-nal | After cardiac surgery | 50 | HES 6% |  | | 500 |  | 15 | 11.6±2.4 | 10.1±2.2 | -1.5 | <0.05 |
| **6** | Skytte Larsson, 2015 [46] | RCT | After cardiac surgery | 15 |  | RA | | 1576^y^ | 20 | 20-30 | 11.2±1.5^c^ | 10.5±1.5^c^ | -0.7 | <0.001 |
|  |  |  |  | 15 | HES 60 mg/ml, 130/0.62 |  | | 783^z^ | 10 | 20-30 | 11.1±1.4^c^ | 9.6±1.3^c^ | -1.5 | <0.001 |
| **7** | Fischer, 2017 [47] | Prospective observation-nal | After cardiac surgery | 11 | HES 130/0.4, VO2 R |  | | 433±103 |  | 30-50 | 11.0±0.8 | 9.7±0.7 | -1.3 | <0.05 |
|  |  |  |  | 6 | HES 130/0.4, VO2 NR |  |  | 409±151 |  | 20-60 | 11.3±1.2 | 10.0±1.2 | -1.3 | <0.05 |
| **8** | Abou-Arab, 2018 [48] | Prospective observation-nal | After cardiovascular surgery | 43 |  | RL FR VO_2_ R | | 500 |  | 10 | 11.4±1.6 | 11.2±1.7 | -0.2 | <0.014^n^ |
|  |  |  |  | 49 |  | RL FR VO_2_ NR | | 500 |  | 10 | 11.2±1.4 | 10.8±1.4 | -0.4 | <0.001^n^ |
| **Circulatory shock / severe hypovolemia** | | | | | | | | | | | | | | |
| **1** | Edwards, 1989 [49] | Interventio-nal | Critically ill patients | 10 | Gelatin 4% |  | | 500 |  | 5 - 10 | 10.9±2.4 | 10.2±1.9 | -0.7 | ns |
| **2** | Bernard, 2003 [50] | Interventio-nal | Post cardiac arrest patients | 22 |  | RL | | 2250^d^ | 30 | 30 | 12.7 [0.12-0.14]^c,h^ | 12.7 [0.11-0.14]^c,h^ | 0 | 0.134 |
| **3** | Meyer, 2008 [51] | Interventio-nal | Hypovolemic shock | 12 | HES 130/0.4 6%, non-sepsis |  | | 500 |  | 15 | 12.1±1.8^c^ | 11.1±1.4^aa^ | -1^bb^ | <0.05 |
|  |  |  |  | 12 | HES 130/0.4 6%, sepsis |  |  | 500 |  | 15 | 10.1±2.4^c^ | 9.3±2.0^aa^ | -0.8^bb^ | <0.05 |
| **4** | Spiel, 2009 [52] | Interventio-nal | Post cardiac arrest patients  Cold solution (4ºC) for TTM | 18 |  | NS or RL | | 2528 | 30 | 30 | 12.9±2.1 | 11.0±1.7^f^ | -1.9 | <0.02 |
| **5** | Monnet, 2013 [53] | Interventio-nal | Acute circulatory failure (78% of septic origin) | 11 |  | NS, FR, VO_2_ NR | | 500 |  | 30 | 10.5±1.5 | 9.7±1.2 | -0.8 | <0.05 |
|  |  |  |  | 14 |  | NS, FR, VO_2_ R | | 500 |  | 30 | 10.5±2.2 | 9.9±2 | -0.6 | <0.05 |
|  |  |  |  | 26 |  | NS, FNR | | 500 |  | 30 | 9.3±1.5 | 8.5±1.5 | -0.8 | <0.05 |
| **6** | Nunes, 2014 [54] | Prospective observation-nal | Circulatory shock | 13 |  | NS or RL, FR | | 500 |  | 30 | 9.4±1.8 | 9.3±2.1 | -0.1 | ns |
|  |  |  |  | 7 |  | NS or RL, FNR | | 500 |  | 30 | 10.0±1.4 | 8.8±1.3 | -1.2 | <0.05 |
| **7** | Guinot, 2016 [55] | Prospective observation-nal | Patients receiving mechanical ventilation with altered tissue perfusion | 31 |  | RL, EtCO_2_ NR | | 500 |  | 10 | 10.9±1.5 | 10.7±1.5 | -0.2 | <0.05 |
|  |  |  |  | 20 |  | RL, EtCO_2_ R | | 500 |  | 10 | 11.8±1.4 | 11.2±1.3 | -0.6 | <0.05 |
| **8** | Mallat, 2016 [56] |  |  | 22 | Albumin 4%, FR, VO2 NR |  | | 500 |  | 15 | 10.7±1.0 | 10.1±0.9 | -0.6 | <0.05 |
|  |  | Prospective observation-nal | Septic shock | 29 | Albumin 4%, FR, VO2 R |  | | 500 |  | 15 | 10.1±1.3 | 9.5±1.1 | -0.6 | <0.05 |
|  |  |  |  | 47 | Albumin 4%, FNR |  | | 500 |  | 15 | 9.3±1.2 | 8.8±1.0 | -0.5 | <0.05 |
| **9** | Xu, 2017 [57] | Prospective observation-nal | Septic shock | 18 | Gelatin 4% or NS or FFP, FR^cc^ | | | 500 |  | 15 | 10.9±3.1 | 9.9±2.7 | -1.0 | 0.003 |
|  |  |  |  | 22 | Gelatin 4% or NS, FNR^dd^ | | | 500 |  | 15 | 9.1±2.0 | 8.5±1.8 | -0.6 | 0.077 |
| **10** | Mongkolpun, 2022 [58] | Prospective observation-nal | Septic shock | 33 |  | | IES, FR | 500 |  | 30 | 10.1±2.0 | 9.9±2.0 | -0.2 | ns |
|  |  |  |  | 29 |  | | IES, FNR | 500 |  | 30 | 9.8±2.5 | 9.5±2.4 | -0.3 | ns |
| **Sepsis** | | | | | | | | | | | | | | |
| **1** | Asfar, 2000 [59] | RCT |  | 16 | HES 6% |  | | 500 |  | 30 | 10.4±2.1 | 9.1±1.8 | -1.3 | 0.001 |
|  |  |  |  | 18 | Gelatin 4% |  |  | 500 |  | 30 | 10.6±1.4 | 9.6±1.4 | -1.0 | 0.0003 |
| **2** | Fernandes, 2001 [60] | RCT |  | 5 | Albumin 5% |  | | 500 |  | 60 | 9.2±0.5 | 9.1±0.8 | -0.1 | ns |
| **3** | Matejovic, 2004 [61] | Interventio-nal |  | 9 | HES 10% |  | | 633±240 |  | 32^ee^ | 9.5 [8.4-9.6]^h^ | 8.2 [6.7-9.6]^h^ | -1.3 | <0.05 |
| **4** | Friedman, 2008 [62] | RCT |  | 11 | HES 10% |  | | 400 |  | 40 | 10.0±1.3 | 8.1±1.1 | -1.9 | <0.05 |
|  |  |  |  | 10 | HES 6% |  |  | 400 |  | 40 | 9.5±1.1 | 8.7±0.9 | -0.8 | ns |
|  |  |  |  | 13 | Albumin 4% |  |  | 400 |  | 40 | 9.6±1.7 | 8.6±1.3 | -1.0 | ns |
| **5** | Smart, 2019 [63] | RCT |  | 24 |  | HS 3% | | 380^n^ |  | 23^n^ | 14.0±1.3^n^ | 13.2±1.2 | -0.8 | <0.0001^n^ |
|  |  |  |  | 25 |  | NS 0.9% | | 1000^n^ |  | 60^n^ | 14.1±1.8^n^ | 13.3±2.0 | -0.8 | 0.0005^n^ |
| **5** | Robba, 2021 [64] | Prospective observation-nal | Mechanical ventilated COVID-19 patients | 22 |  | “Crystalloid” | | 280^ff^ | 4 | 20 | 7.7 [7.1-8.8]^h^ | 8.5 [8.1-8.8]^h^ | 0.8 | 0.434 |
| **Mixed conditions** | | | | | | | | | | | | | | |
| **1** | Spoelstra-de Man, 2017 [65] | RCT | Cardiac surgery (n = 39);  Major vascular surgery (n = 28); other major surgery and trauma (n = 24); sepsis (n = 24) | 29 | HES 60 g/L, 200/0.45-0.55 |  | | 1441±295 |  | 90 | 9.2±1.8^gg^ | 8.1±1.3^gg^ | -1.1 | <0.001 |
|  |  |  |  | 30 | Albumin 50 g/L, 20% |  |  | 1553±258 |  | 90 | 9.5±1.9^gg^ | 8.4±1.4^gg^ | -1.1 | <0.001 |
|  |  |  |  | 28 | Gelatin 40 g/L |  |  | 1509±328 |  | 90 | 9.2±1.4^gg^ | 8.1±1.1^gg^ | -1.1 | <0.001 |
|  |  |  |  | 28 |  | NS | | 1723±209 |  | 90 | 10±1.9^gg^ | 9.8±1.4^gg^ | -0.2 | <0.001 |

Data given as mean ± SD or median [IQR]; IES: isotonic electrolyte solution; NS: normal saline; RL: Ringer’s lactate; HES: hydroxyethyl starch; HS: hypertonic solution; Hct: hematocrit; ns: not significant; S: significant; FFP: fresh frozen plasma; RA: Ringer acetate; RCT: randomized controlled trial; FR: fluid responder; FNR: fluid non-responder; VO_2_R: oxygen consumption responders; VO_2_NR: oxygen consumption non-responders; NR: not reported in the original paper; EtCO2 NR: end-tidal carbon dioxide tension non-responders; EtCO2 R: end-tidal carbon dioxide tension responders

**^a^** This article was reported in the paper of Ehrly, 1985, however the original manuscript was not retrievable.

**^b^** not included in meta-analysis because both SD, IQR or SEM of Hb values and p values were not reported.

**^c^** Hb calculated from Hct.

**^d^** Weight assumed 75 kg.

**^e^** Data given as mean ± SEM.

**^f^** Values obtained from the reported graphs

**^g^** Calculated based on a reported mean weight of 76.4 Kg with 15 mL/Kg of fluid administered

**^h^** Data presented as median [IQR]

**^i^** Value calculated from the 7.5% decrease reported

**^j^** Calculated from a body surface area of 1.92 (assumed from a weight of 75 Kg and a height of 1.75m

**^k^** Values taken from the percentage change in Hb from baseline

**^l^** Calculated based on a reported mean weight of 76.4 Kg

**^m^** Infusion time was 3-5 minutes. In between boluses there was a 5-minute pause. We report the average time (4 minutes) multiplied by the number of boluses given plus the 5-minute pause between each fluid administration

**^n^** Information provided by the author

**^o^** p calculated for MA according to Hb data provided by the authors

**^p^** Calculated based on a reported mean weight of 80 Kg

**^q^** Calculated based on a reported mean weight of 87 Kg

**^r^** Infusion time was 2-3minutes. In between boluses there was a 5-minute pause. We report the average time (2.5 minutes) multiplied by the number of boluses given plus the 5-minute pause between each fluid administration

**^s^** RL+HES was given a loading dose of 10 ml/kg RL over 20 min, followed by 6% HES over 30min

**^t^** Calculated from a weight of 77 Kg

**^u^** Bolus amounts calculated according to the 1000 mL of total infusion after 10 boluses

**^v^** Each bolus was infused in 10 equally divided doses administered over 1-hour

**^w^** Several boluses with the same amount to a total of 40 mL/kg after 10 boluses, for a weight of 67.5 Kg

**^x^** Calculated based on a reported body weight of 78.4 Kg with 30 mL/Kg of fluid administered

**^u^** Calculated based on a reported mean weight of 78.8 Kg with 20 mL/Kg of fluid administered

**^z^** Calculated based on a reported mean weight of 78.3 Kg with 10 mL/Kg of fluid administered

**^aa^** Calculated from the absolute reduction in hematocrit

**^bb^** The mean difference of the SD reported was used for the meta-analysis

**^cc^** Fluid chosen at discretion of treating physician - 16 patients received gelatin, 1 FFP and 1 NS

**^dd^** Fluid chosen at discretion of treating physician - 15 patients received gelatin and 7 NS

**^ee^** Calculated considering 200 mL given every 10 minutes for a total of 633mL

**^ff^** Calculated based on a reported predicted body weight of 70 Kg with 4 mL/Kg of fluid administered

**^gg^**Hb calculated from mmol/L

# Table E3. Description of studies and study sets by group and subgroup for Hb decrease and DO_2_

|  | **Total non-acutely ill** | **Acutely ill** | | | | | **ALL** |
| --- | --- | --- | --- | --- | --- | --- | --- |
|  |  | **Sepsis** | **Circulatory shock/severe hypovolemia** | **Surgery/**  **trauma** | **Mixed conditions** | **Total acutely ill** |  |
| ***Hb decrease*** | | | | | | | |
| Total studies (n) | 40 | 6 | 10 | 8 | 1 | 25 | 65 |
| RCT | 23 | 4 | 0 | 2 | 1 | 7 | 30 |
| Non-RCT | 17 | 2 | 10 | 6 | 0 | 18 | 35 |
| Total study sets (n) | 104 | 10 | 19 | 20 | 4 | 53 | 157 |
| MA studies (n) | 38 | 6 | 10 | 8 | 1 | 25 | 63 |
| MA study sets | 101 | 10 | 19 | 20 | 4 | 53 | 154 |
| Infusion duration (min)^a, b^ | 30 | 36 | 15 | 40 | 90 | 30 | – |
| **Colloids** | | | | | | | |
| Total study sets (n) | 51 | 7 | 6 | 16 | 3 | 32 | 83 |
| MA study sets | 50 | 7 | 6 | 16 | 3 | 32 | 82 |
| Amount of fluid (mL)^c, b^ | 735 | 476 | 500 | 583 | 1501 | 630 | – |
| **Crystalloids** | | | | | | | |
| Total study sets (n) | 46 | 3 | 11 | 4 | 1 | 19 | 65 |
| MA study sets | 44 | 3 | 11 | 4 | 1 | 19 | 63 |
| Amount of fluid (mL)^c, b^ | 1366 | 553 | 843 | 894 | 1723 | 855 | – |
| **Mixed solutions^d^** | | | | | | | |
| **Total (not MA included) study sets (n)** | 7 | 0 | 2 | 0 | 0 | 2 | 9 |
| **Duration of administration** | | | | | | | |
| **≤1 h study sets for MA (n)** | 100 | 10 | 19 | 18 | 0 | 47 | 147 |
| **>1 h study sets for MA (n)** | 1 | 0 | 0 | 2 | 4 | 6 | 7 |
| ***DO_2_*** | | | | | | | |
| Total studies (n) | 1^e^ | 3 | 6 | 5 | 0 | 14 | 15 |
| Total study sets (n) | 1^e^ | 6 | 12 | 9 | 0 | 27 | 28 |
| MA studies (n) | 0 | 3 | 6 | 4 | 0 | 13 | 13 |
| MA study sets | 0 | 6 | 11 | 7 | 0 | 24 | 24 |

MA: meta-analysis; n: count

^a^data presented as median.

^b^taken from MA study sets

^c^data presented as mean

^d^colloids and crystalloids

^e^pre-surgical setting

# Figure E1. Complete Forest plot with all the included studies


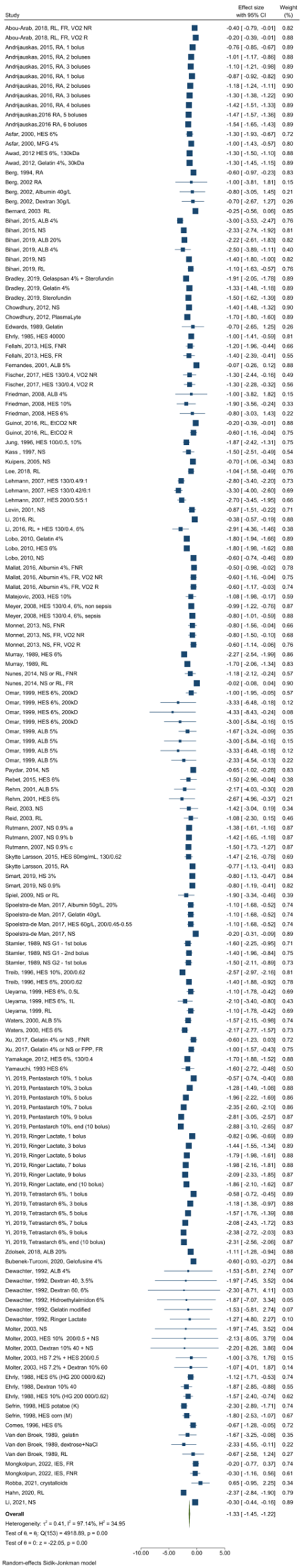


#

# Table E4. Change in hemoglobin concentration (Hb) after rapid fluid administration in the different study groups

|  | **Delta Hb** | **95% CI** | **p*** | **p**** | **p***** | **p****** | **p******* | **I^2^** |
| --- | --- | --- | --- | --- | --- | --- | --- | --- |
| **OVERALL** | -1.33 | [-1.45 – -1.12] | - | - | - | - | - | 96.88 |
| **Non-acutely ill** | -1.56 | [-1.69 – -1.42] | - | 0.138 | **0.003** | **<0.001** | **<0.001** | 96.71 |
| **Acutely ill** | -0.84 | [-1.03 – -0.64] | **<0.001** | - | - | **-** | **-** | 92.91 |
| Sepsis | -0.78 | [-1.18 – -0.37] | **<0.001** | 0.121 | 0.931 | - | 0.358 | 78.36 |
| Circulatory shock/severe hypovolemia | -0.57 | [-0.75 – -0.38] | **<0.001** | **0.004** | 0.366 | 0.358 | - | 78.71 |
| Surgery/trauma | -1.23 | [-1.64 – -0.82] | 0.138 | - | 0.182 | 0.121 | **0.004** | 90.00 |
| Mixed conditions | -0.80 | [-1.28 – -0.33] | **0.003** | 0.182 | - | 0.931 | 0.366 | 78.94 |

*Compared to non-acutely ill; **compared to the ‘surgery/trauma’ subgroup; ***compared to the ‘mixed conditions’ subgroup, ****compared to the ‘sepsis’ subgroup, *****compared to circulatory shock/severe hypovolemia

# Figure E2. Meta-regression for fluid duration in non-acutely ill


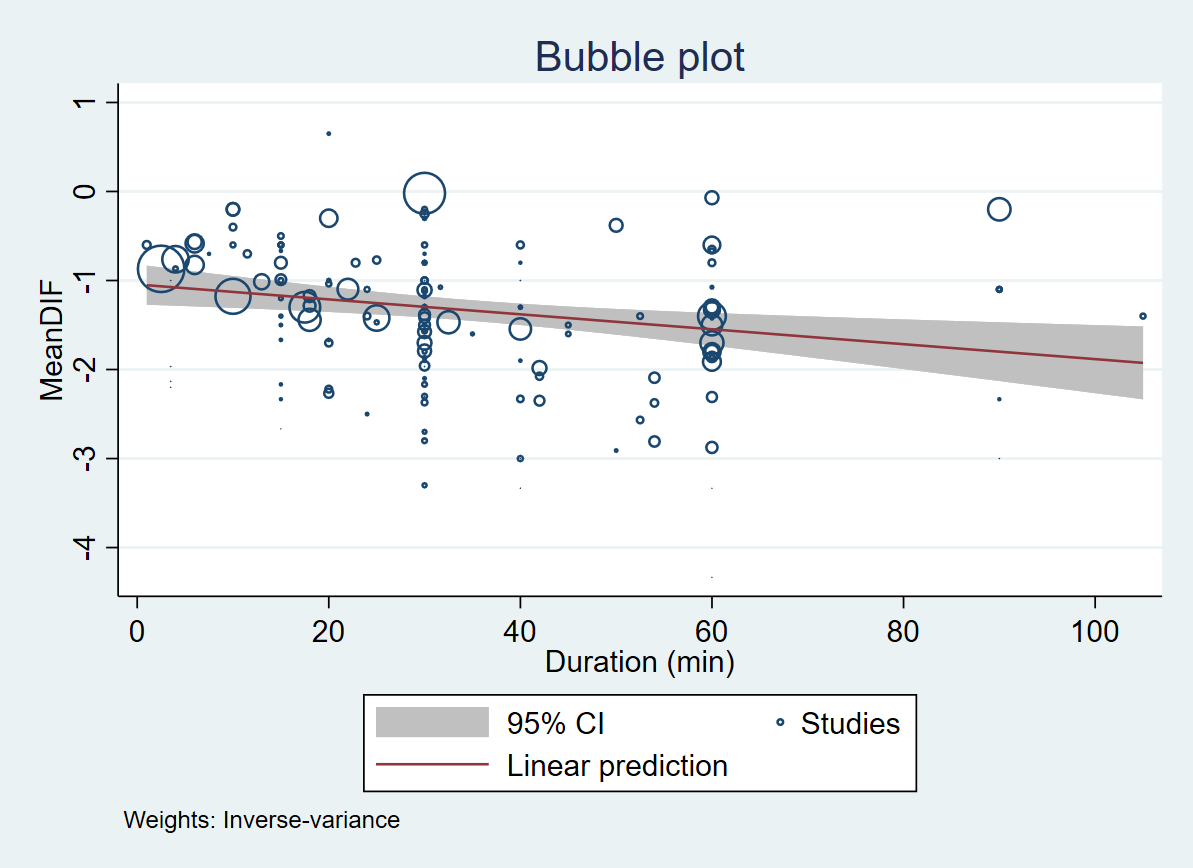


# Figure E3. Metaregression for fluid amount in non-acutely ill


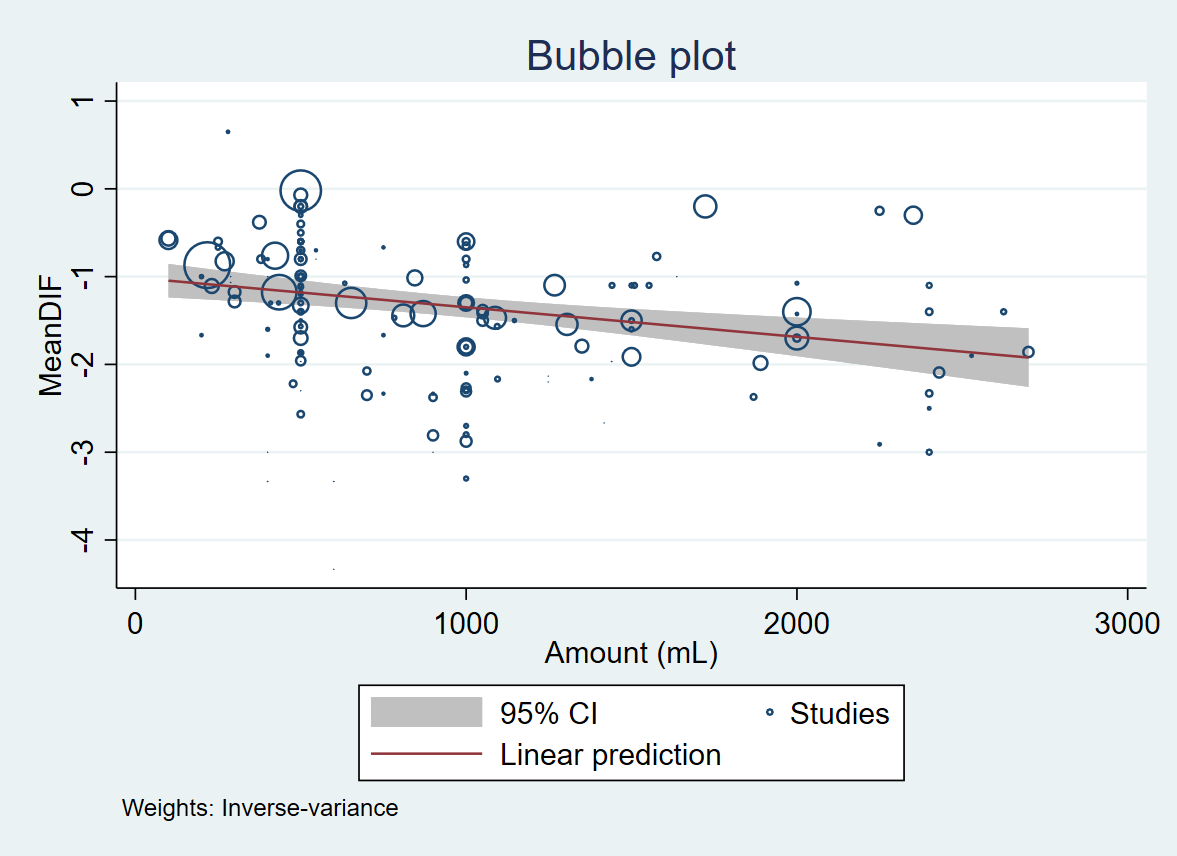


# Figure E4. Change in hemoglobin concentration (Hb) from baseline in the four subgroups of acutely ill patients (circulatory shock/severe hypovolemia, sepsis, surgery/trauma, and mixed conditions) according to rate of fluid administration, type of fluid, baseline hemoglobin and fluid responsiveness.

**
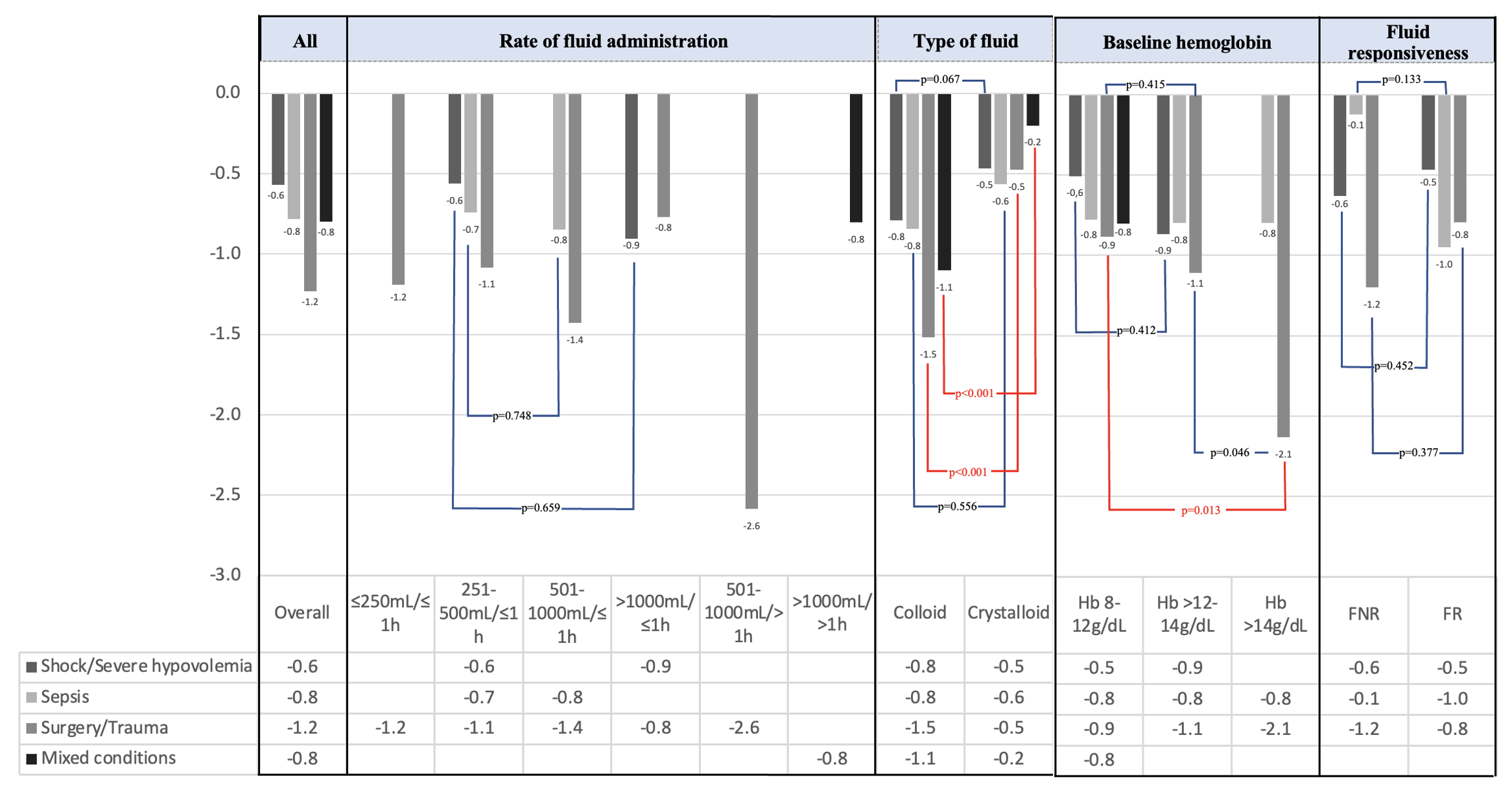
**

FR: fluid responder; FNR: fluid non-responder

# Figure E5. Decrease in hemoglobin concentration (Hb) in non-acutely ill and acutely ill patients according to rate of fluid administration


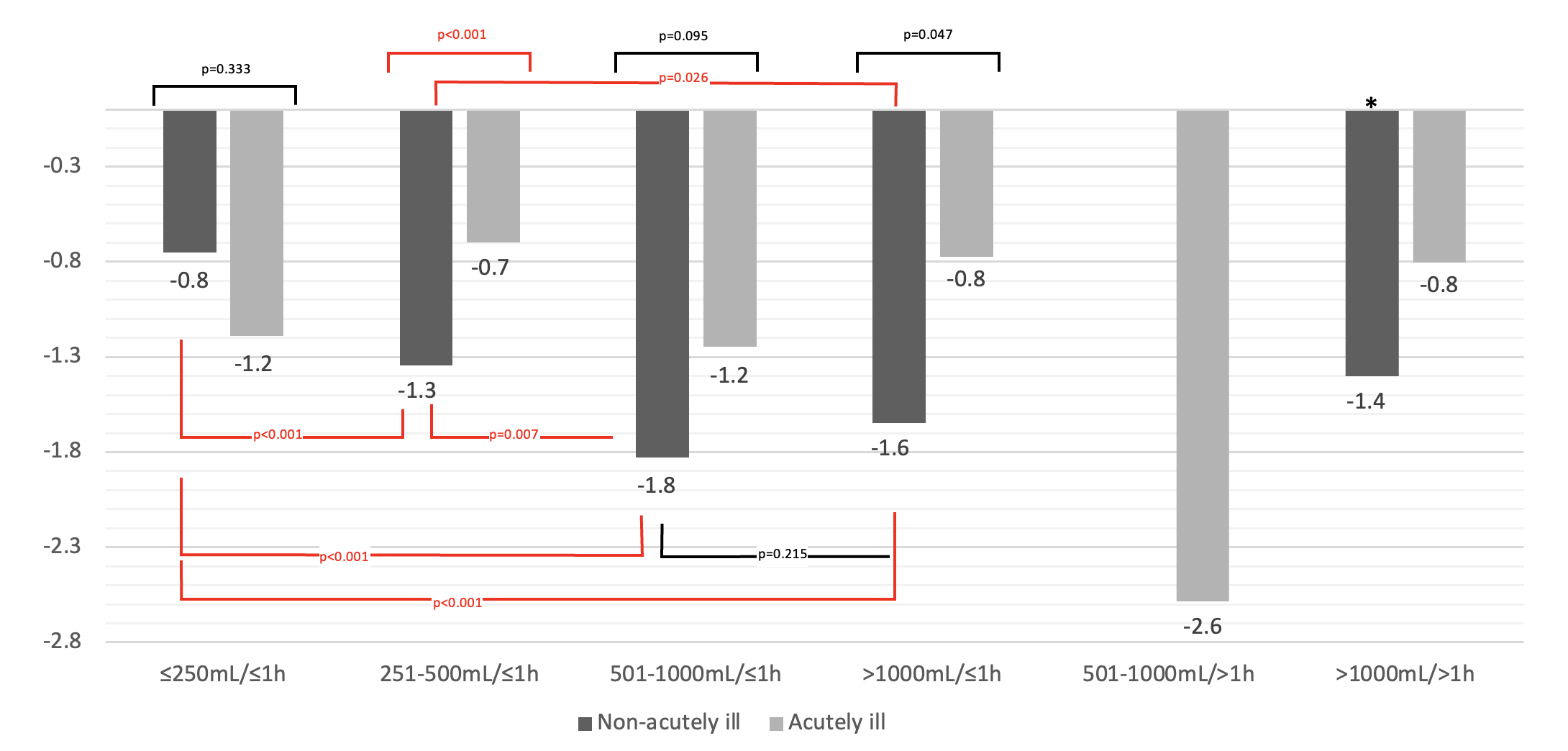


*Only one study set of ten patients (Stamler, 1989)

# Figure E6. Change in hemoglobin concentration (Hb) from baseline in acutely ill patients at different time points


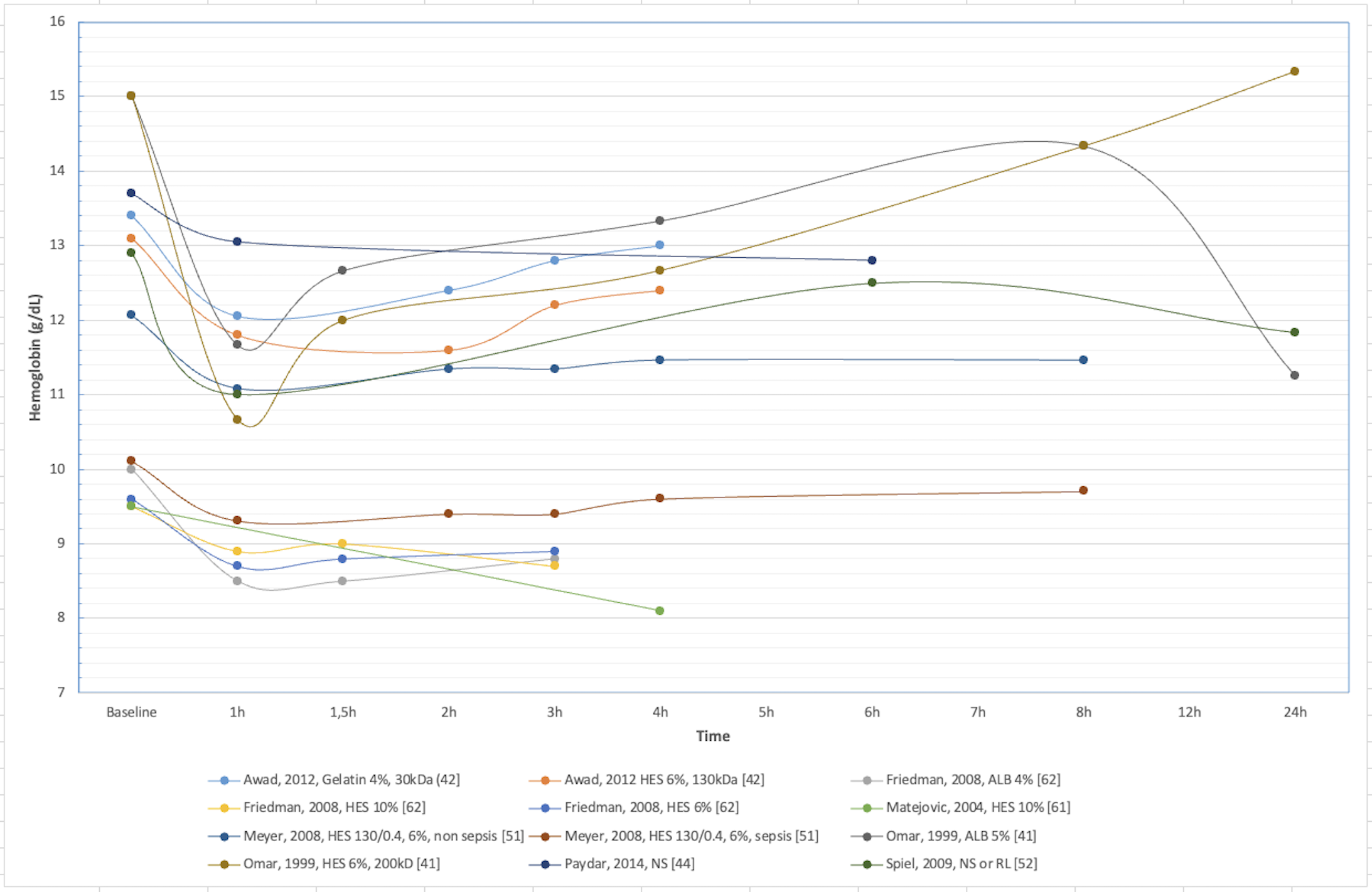


# Table E5. Delta hemoglobin (Hb) according to type of fluid

|  | **Delta Hb** | **CI 95%** | **p*** | **I^2^** |
| --- | --- | --- | --- | --- |
| **OVERALL** | | | |  |
| Colloid | -1.569 | [-1.738 – -1.400] | <0.001 | 92.18 |
| Crystalloid | -1.065 | [-1.213 – -0.916] |  | 97.69 |
| **NON-ACUTELY ILL** | | | |  |
| Colloid | -1.799 ^a,b,c,d,e^ | [-2.001 – -1.598] | <0.001 | 92.94 |
| Crystalloid | -1.299 ^f,g,h,i,j^ | [-1.445 – -1.153] |  | 96.86 |
| **ACUTELY ILL** | | | |  |
| Colloid | -1.115^e^ | [-1.378 – -0.852] | <0.001 | 84.50 |
| Crystalloid | -0.473^f^ | [-0.671 – -0.276] |  | 89.48 |
| **CIRCULATORY SHOCK/SEVERE HYPOVOLEMIA** | | | |  |
| Colloid | -0.789^b^ | [-0.974 – -0.604] | 0.067 | 26.71 |
| Crystalloid | -0.466^g^ | [-0.757 – -0.175] |  | 84.93 |
| **SEPSIS** | | | |  |
| Colloid | -0.843^c^ | [-1.304 – -0.382] | 0.556 | 64.88 |
| Crystalloid | -0.567^h^ | [-1.360 – 0.225] |  | 84.88 |
| **SURGERY/TRAUMA** | | | |  |
| Colloid | -1.518^d^ | [-1.951 – -1.084] | <0.001 | 75.77 |
| Crystalloid | -0.475^i^ | [-0.739 – -0.211] |  | 63.72 |
| **MIXED CONDITIONS** | | | |  |
| Colloid | -1.100^e^ | [-1.435 – -0.765] | <0.001 | 0.00 |
| Crystalloid | -0.200^j^ | [-0.305 – -0.095] |  | 0.00 |

p value: colloid versus crystalloid

^f,g,i,j^p<0.001 in crystalloid between non-acutely ill vs.: acutely ill, shock/severe hypovolemia, sepsis, surgery/trauma and ‘mixed conditions’, respectively.

^h^p=0.075 in crystalloid between non-acutely ill vs. sepsis

^a,b,c,e^p<0.001 in colloid between non-acutely ill vs.: acutely ill, circulatory shock/severe hypovolemia, sepsis, ‘mixed conditions’, respectively.

^d^p=0.248 in colloid between non-acutely ill vs. surgery/trauma.

# Table E6. Delta hemoglobin (Hb) by ‘amount of fluid (mL) over ≤1 h’

|  | **Delta Hb** | **CI 95%** | **p** | **I^2^** |
| --- | --- | --- | --- | --- |
| **NON-ACUTELY ILL** | | |  |  |
| ≤250 | -0.751^a,b,c,d^ | [-0.932 – -0.569] | <0.001 | 87.04 |
| 251-500 | -1.344^a,e,f,g^ | [-1.544 – -1.144] |  | 92.44 |
| 501-1000 | -1.828^b,e,h,i^ | [-2.119 – -1.537] |  | 97.40 |
| >1000 | -1.648^c,f,h,j^ | [-1.826 – -1.470] |  | 93.30 |
| **ACUTELY ILL** | | |  |  |
| ≤250 | -1.191^d^ | [-2.063 – -0.319] | 0.340 | 9.27 |
| 251-500 | -0.698^g^ | [-0.907 – -0.489] |  | 89.07 |
| 501-1000 | -1.248^i^ | [-1.910 – -0.586] |  | 94.09 |
| >1000 | -0.774^j^ | [-1.617 – -0.068] |  | 88.60 |
| **CIRCULATORY SHOCK/SEVERE HYPOVOLEMIA** | | |  |  |
| 251-500 | -0.562 | [-0.732 – -0.392] | 0.659 | 71.66 |
| >1000 | -0.905 | [-2.418 – -0.608] |  | 77.37 |
| **SEPSIS** | | |  |  |
| 251-500 | -0.741 | [-1.261 – -0.221] | 0.748 | 82.17 |
| 501-1000 | -0.846 | [-1.223 – -0.469] |  | 3.7 |
| **SURGERY/TRAUMA** | | |  |  |
| ≤250 | -1.191 | [-2.063 – -0.319] | 0.466 | 9.27 |
| 251-500 | -1.083 | [-1.676 – -0.490] |  | 81.78 |
| 501-1000 | -1.429 | [-2.320 – -0.538] |  | 95.82 |
| >1000 | -0.770 | [-1.130 – -0.410] |  | 0 |

^a,b,c,g^p<0.001 in non-acutely ill between ≤250 mL/≤1 h vs. 251-500 mL/≤1 h, 501-1000 mL/≤1 h, >1000 mL/≤1 h; non-acutely ill 251-500 mL/≤1 h vs. acutely-ill 251-500 mL/≤1 h, respectively.

^e^p=0.007 in non-acutely ill between 251-500 mL/≤1 h vs. 501-1000 mL/≤1 h.

^f^p=0.026 in non-acutely ill between 251-500 mL/≤1 h vs. >1000 mL/≤1 h.

^h^p=0.215 in non-acutely ill between 501-1000 mL/≤1 h vs. >1000 mL/≤1 h.

^d^p=0.333 in ≤250 mL/≤1 h between non-acutely ill vs. acutely ill.

^i^p=0.095 in 501-1000 mL/≤1 h between non-acutely ill vs. acutely ill.

^j^p=0.047 in >1000 mL/≤1 h between non-acutely ill vs. acutely ill.

# Table E7. Delta Hb by ‘amount of fluid (mL) over >1h’

|  | **Delta Hb** | **CI 95%** | **p** | **I^2^** |
| --- | --- | --- | --- | --- |
| **NON-ACUTELY ILL** | | |  |  |
| >1000 | -1.400 | [-1.965 – -0.835] |  | 0 |
| **ACUTELY ILL** | | |  |  |
| 501-1000 | -2.585 | [-4.334 – -0.836] | 0.054 | 0.81 |
| >1000 | -0.803 | [-1.278 – -0.328] |  | 78.94 |
| **MIXED CONDITIONS** | | |  |  |
| >1000 | -0.803 | [-1.278 – -0.328] | - | 78.94 |
| **SURGERY/TRAUMA** | | | |  |
| 501-1000 | -2.585 | [-4.334 – -0.836] | - | 0.81 |

# Table E8. Delta hemoglobin (Hb) for different baseline Hb categories

|  | **Delta Hb** | **CI 95%** | **p** | **I^2^** |
| --- | --- | --- | --- | --- |
| **NON-ACUTELY ILL** | | | |  |
| Hb 8-12 g/dL | -1.416^a,b,c,d^ | [-1.946 – -0.886] | 0.021 | 75.46 |
| Hb >12-14 g/dL | -1.354^e,b,f,g^ | [-1.510 – -1.197] |  | 96.16 |
| Hb >14 g/dL | -1.708^h,c,f,i^ | [-1.903 – -1.512] |  | 95.63 |
| **ACUTELY ILL** | | | |  |
| Hb 8-12 g/dL | -0.685^j,k,a^ | [-0.849 – -0.522] | 0.018 | 85.93 |
| Hb >12-14 g/dL | -0.939^j,l,e^ | [-1.286 – -0.591] |  | 89.24 |
| Hb >14 g/dL | -1.841^k,l,h^ | [-2.694 – -0.988] |  | 55.02 |
| **CIRCULATORY SHOCK/SEVERE HYPOVOLEMIA** | | | |  |
| Hb 8-12 g/dL | -0.507 | [-0.680 – -0.335] | 0.412 | 66.18 |
| Hb >12-14 g/dL | -0.866 | [-1.705 – -0.027] |  | 92.03 |
| **SEPSIS** | | | |  |
| Hb 8-12 g/dL | -0.777 | [-1.323 – -0.230] | 0.997 | 75 |
| Hb >12-14 g/dL | -0.800 | [-1.131 – -0.469] |  | 0 |
| Hb >14 g/dL | -0.800 | [-1.190 – -0.410] |  | 0 |
| **SURGERY/TRAUMA** | | | |  |
| Hb 8-12 g/dL | -0.885^d,m,n^ | [-1.238 – -0.532] | 0.044 | 70.69 |
| Hb >12-14 g/dL | -1.108^g,m,o^ | [-1.513 – -0.704] |  | 86.60 |
| Hb >14 g/dL | -2.130^i,n,o^ | [-3.048 – -1.213] |  | 28.41 |
| **MIXED CONDITIONS** | | | |  |
| Hb 8-12 g/dL | -0.803 | [-1.278 – -0.328] | – | 78.94 |

^j^p=0.196 in acutely ill between baseline Hb 8-12 g/dL vs. >12-14 g/dL

^k^p=0.009 in acutely ill between baseline Hb 8-12 g/dL vs. >14 g/dL

^l^p=0.055 in acutely ill between baseline Hb >12-14 g/dL vs. >14 g/dL

^a^p=0.010 in baseline Hb 8-12 g/dL between acutely ill vs. non-acutely ill

^e^p=0.033 in baseline Hb >12-14 g/dL between acutely ill vs. non-acutely ill

^h^p=0.765 in baseline Hb >14 g/dL between acutely ill vs. non-acutely ill

^b^p=0.825 in non-acutely ill between baseline Hb 8-12 g/dL vs. >12-14 g/dL

^c^p=0.312 in non-acutely ill between baseline Hb 8-12 g/dL vs. >14 g/dL

^f^p=0.006 in non-acutely ill between baseline Hb >12-14 g/dL vs. >14 g/dL

^d^p=0.102 in baseline Hb 8-12 g/dL between acutely ill vs. surgery/trauma subgroup

^g^p=0.267 in baseline Hb >12-14 g/dL between acutely ill vs. surgery/trauma subgroup

^i^p=0.377 in baseline Hb >14 g/dL between acutely ill vs. surgery/trauma subgroup

^m^p=0.415 in surgery/trauma subgroup between baseline Hb 8-12 g/dL vs. >12-14 g/dL

^n^p=0.013 in surgery/trauma subgroup between baseline Hb 8-12 g/dL vs. >14 g/dL

^o^p=0.046 in surgery/trauma subgroup between baseline Hb >12-14 g/dL vs. >14 g/dL

# Table E9. Delta hemoglobin (Hb) in fluid responders (FR) and non-responders (FNR)

|  | **Delta Hb** | **CI 95%** | **p** | **I^2^** |
| --- | --- | --- | --- | --- |
| **ACUTELY ILL** | | |  |  |
| FR | -0.683 | [-0.941 – -0.425] | 0.510 | 83.10 |
| FNR | -0.548 | [-0.854 – -0.241] |  | 44.64 |
| **CIRCULATORY SHOCK/SEVERE HYPOVOLEMIA** | | |  |  |
| FR | -0.467 | [-0.705 – -0.228] | 0.452 | 52.37 |
| FNR | -0.629 | [-0.981 – -0.278] |  | 21.66 |
| **SEPSIS** | | |  |  |
| FR | -0.950 | [-1.924 – 0.024] | 0.133 | 80.20 |
| FNR | -0.125 | [-0.587 – 0.338] |  | 5.94 |
| **SURGERY/TRAUMA** | | |  |  |
| FR | -0.793 | [-1.285 – -0.301] | 0.377 | 72.20 |
| FNR | -1.200 | [-1.956 – -0.444] |  | 0 |

# Figure E7. Change in hemoglobin concentration (Hb) from baseline in non-acutely ill subjects at different time points


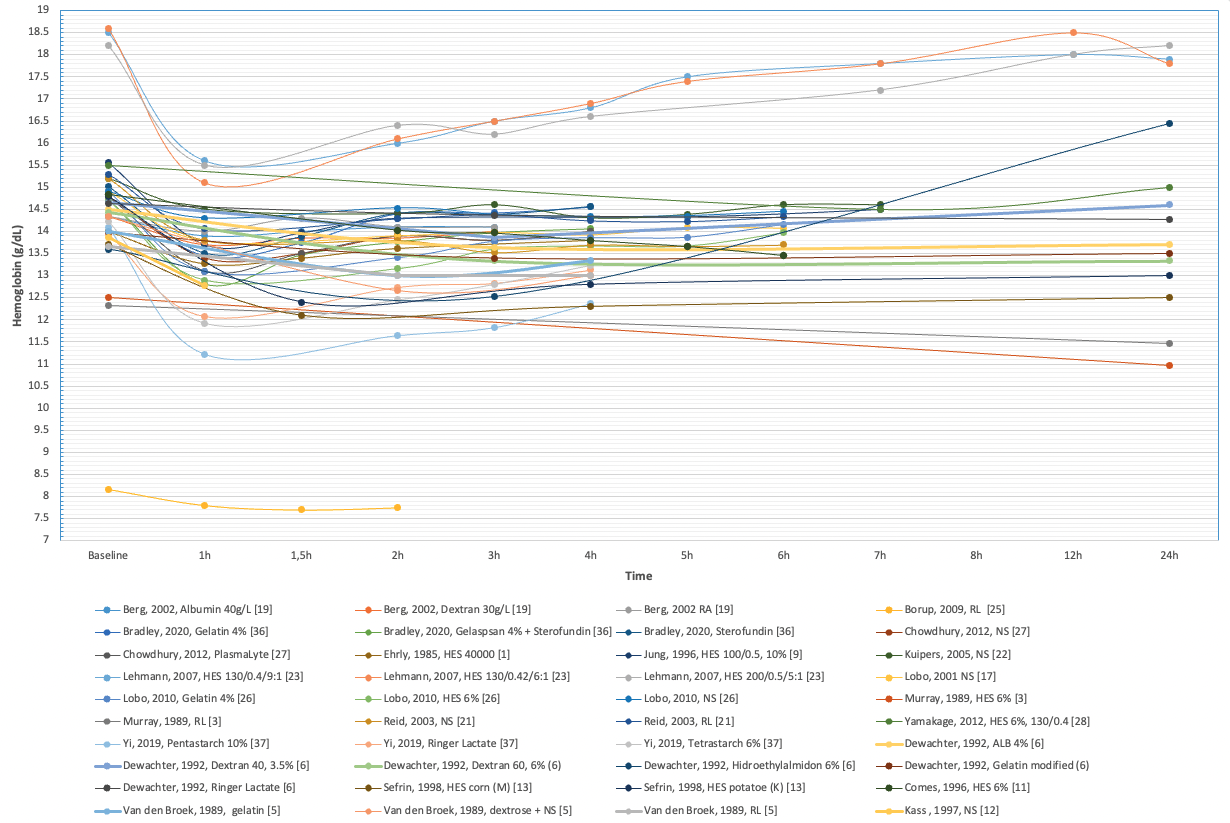


# Table E10. Clinical studies that reported fluid responsiveness as defined by change in cardiac index (CI)

| **First author, year** | **Fluid type** | **Amount of fluid (mL)** | **Duration of infusion (min)** | **Initial CI**  **(L/min/m^2^)** | **Post-fluid CI**  **(L/min/m^2^)** | **P value** | **Initial**  **DO_2_I**  **(ml/min/ m^2^)** | **Post-fluid DO_2_I**  **(ml/min/m^2^)** | **P value** |
| --- | --- | --- | --- | --- | --- | --- | --- | --- | --- |
| Edwards, 1989 [49] | Gelatin 4% | 500 | 5 - 10 | 3.4±1.6 | 4.1±1.7 | <0.05 | 455±219 | 525±234 | NR |
| Asfar, 2000 [59] | HES 6% | 500 | 30 | 3.7±1.1 | 4.4±0.9 | 0.012 | 481±110 | 514±134 | ns |
|  | Gelatin 4% | 500 | 30 | 3.6±1.1 | 4.4±1.4 | 0.006 | 498±140 | 531±140 | ns |
| Fernandes, 2001 [60] | Albumin 5% | 500 | 60 | 4.9±1.1 | 5.2±1.3 | ns | 700±183 | 732±195 | ns |
| Friedman, 2008 [62] | HES 10% | 400 | 40 | 3.2±1.2 | 3.8±1.2 | NR | 415±152 | 416±127 | NR |
|  | HES 6% | 400 | 40 | 3.7±0.8 | 3.8±0.8 | NR | 472±110 | 451±95 | NR |
|  | Albumin 4% | 400 | 40 | 3.8±0.8 | 3.8±1.0 | NR | 454±119 | 416±92 | NR |
| Fellahi, 2013 [43] | HES 130/0.4 6% (FR) | 500 | 15 | 2.2±0.5 | 2.8±0.6 | <0.01 | 348±105 | 395±114 | <0.01 |
|  | HES 130/0.4 6% (FNR) | 500 | 15 | 2.1±0.4 | 2.2±0.4 | ns | 320±84 | 299±60 | <0.01 |
| Monnet, 2013 [53] | NS, FR, VO_2_ NR | 500 | 30 | 2.7±1.1 | 3.7±1.4 | <0.05 | 382±202 | 488±234 | <0.05 |
|  | NS, FR, VO_2_ R | 500 | 30 | 2.7±1.2 | 3.7±1.5 | <0.05 | 388±197 | 502±229 | <0.05 |
|  | NS, FNR | 500 | 30 | 3.6 ±1.4 | 3.7±1.5 | ns | 436±158 | 418±154 | <0.05 |
| Nunes, 2014 [54] | NS or RL, FR | 500 | 30 | 2.8±0.6 | 3.6±0.7 | <0.05 | NR | NR | NR |
|  | NS or RL, FNR | 500 | 30 | 3.4±0.6 | 3.6±0.7 | ns | NR | NR | NR |
| Rebet, 2015 [45] | HES 6% | 500 | 15 | 2.2±0.5 | 2.7±0.6 | <0.05 | 340±100 | 370±111 | ns |
| Skytte Larsson, 2015 [46]^a^ | HES 60 mg/ml, 130/0.62 | 783^b^ | 20 - 30 | 2.6±0.6 | 3.2±0.5 | NR | 387±92 | 406±77 | NR |
|  | RA | 1567^c^ | 20 - 30 | 2.5±0.5 | 2.9±0.7 | NR | 365±73 | 400±89 | NR |
| Guinot, 2016 [55] | RL, EtCO_2_ NR^a^ | 500 | 10 | 1.8 [1.5-2.6]^d^ | 2.3 [1.8 - 2.8]^d^ | <0.05 | 251 [188 – 326]^d^ | 295 [192 – 373]^d^ | <0.05 |
|  | RL, EtCO_2_ R | 500 | 10 | 1.8 [1.3 – 2.4]^d^ | 2.4 [1.6 – 3.4]^d^ | <0.05 | 283 [204 – 377]^d^ | 373 [251 – 452]^d^ | <0.05 |
| Mallat, 2016 [56]^e^ | Albumin 4%, FR, VO_2_ NR | 500 | 15 | 3.3 [2.5 – 3.7] | 3.8 [3.5 – 4.2] | <0.05 | 409±80 | 529±84 | <0.05 |
|  | Albumin 4%, FR,  VO_2_ R | 500 | 15 | 2.6 [1.4 – 3.0] | 3.0 [2.1 – 3.7] | <0.05 | 297±86 | 381±84 | <0.05 |
| Xu, 2017 [57] | Gelatin 4% or NS or FFP, FR | 500 | 15 | 3.4±1.1 | 4.4±1.0 | <0.001 | 448±139 | 550±126 | <0.001 |
|  | Gelatin 4% or NS, FNR | 500 | 15 | 4.1±1.3 | 4.1±1.3 | 0.801 | 500±232 | 461±194 | 0.159 |
| Fischer, 2017 [47] | HES 130/0.4, VO_2_ R | 433±103 | 30 - 50 | 1.3±0.4 | 2.4±0.7 | <0.05 | 193±60 | 313±96 | <0.05 |
|  | HES 130/0.4, VO_2_ NR | 409±151 | 20 - 60 | 1.7±0.4 | 2.7±1.0 | <0.05 | 285±49 | 389±139 | <0.05 |
| Abou-Arab, 2018 [48] | RL, FR VO_2_ R | 500 | 10 | 1.7±0.6 | 2.3±0.7 | <0.05 | 269±103 | 339±124 | <0.05 |
|  | RL, FR VO_2_ NR | 500 | 10 | 1.8±0.7 | 2.2±0.9 | <0.05 | 274±95 | 319±119 | <0.05 |
| Bubenek-Turconi, 2020 [38]^a^ | Gelatin 4% | 250 | 1 | 2.1±0.4^f^ | 2.3±0.5^f^ | 0.001 | 354±88^f^ | 376±78^f^ | 0.006 |
| Mongkolpun, 2022 [58] | IES, FR | 500 | 30 | 2.3±1.2 | 3.1±1.2 | <0.05 | 249 [173-386] | 337 [252-424] | <0.05 |
|  | IES, FNR | 500 | 30 | 2.4±0.7 | 2.6±0.8 | <0.05 | 308 [229-375] | 308 [211-353] | ns |

Data given as mean ± SD or median [IQR]; FNR: fluid non-responder; FR: fluid responder; HES: hydroxyethyl starch; IES: isotonic electrolyte solution; NR: not reported in the original paper; NR: not reported; ns: non-significant; NS: normal saline; RL: Ringer’s lactate; VO2 NR: oxygen consumption non-responders; VO2 R: oxygen consumption responders.

^a^not included in the meta-analysis either for CI or DO2, because this study had mixed FR and FNR patients

^b^calculated from 10 ml/Kg and reported weight of 78.3 Kg

^c^calculated from 20 ml/Kg and reported weight of 78.8 Kg

^d^calculated from a body surface area of 1.92 (assumed from a weight of 75 Kg and a height of 1.75 m)

^e^the FNR study set is not described because CI data were not reported

**^f^**calculated from a reported weight of 78.6 Kg and a height of 171.9 cm.

# Table E11. Delta oxygen delivery (DO_2_) in fluid responders (FR) and non-responders (FNR)

|  | **Delta DO_2_** | **CI 95%** | **P** | **I^2^** |
| --- | --- | --- | --- | --- |
| **ACUTELY ILL** | | |  |  |
| Overall | 35.797 | [13.395 – 58.199] | <0.001 | 82.47 |
| FR | 67.757 | [46.114 – 89.400] |  | 27.49 |
| FNR | -16.304 | [-31.515 – -1.093] |  | 55.28 |

# Table E12. Changes in oxygen delivery (DO_2_) according to change in cardiac index (CI) in studies showing statistically significant (left) and non-significant (right) decrease in hemoglobin (Hb)


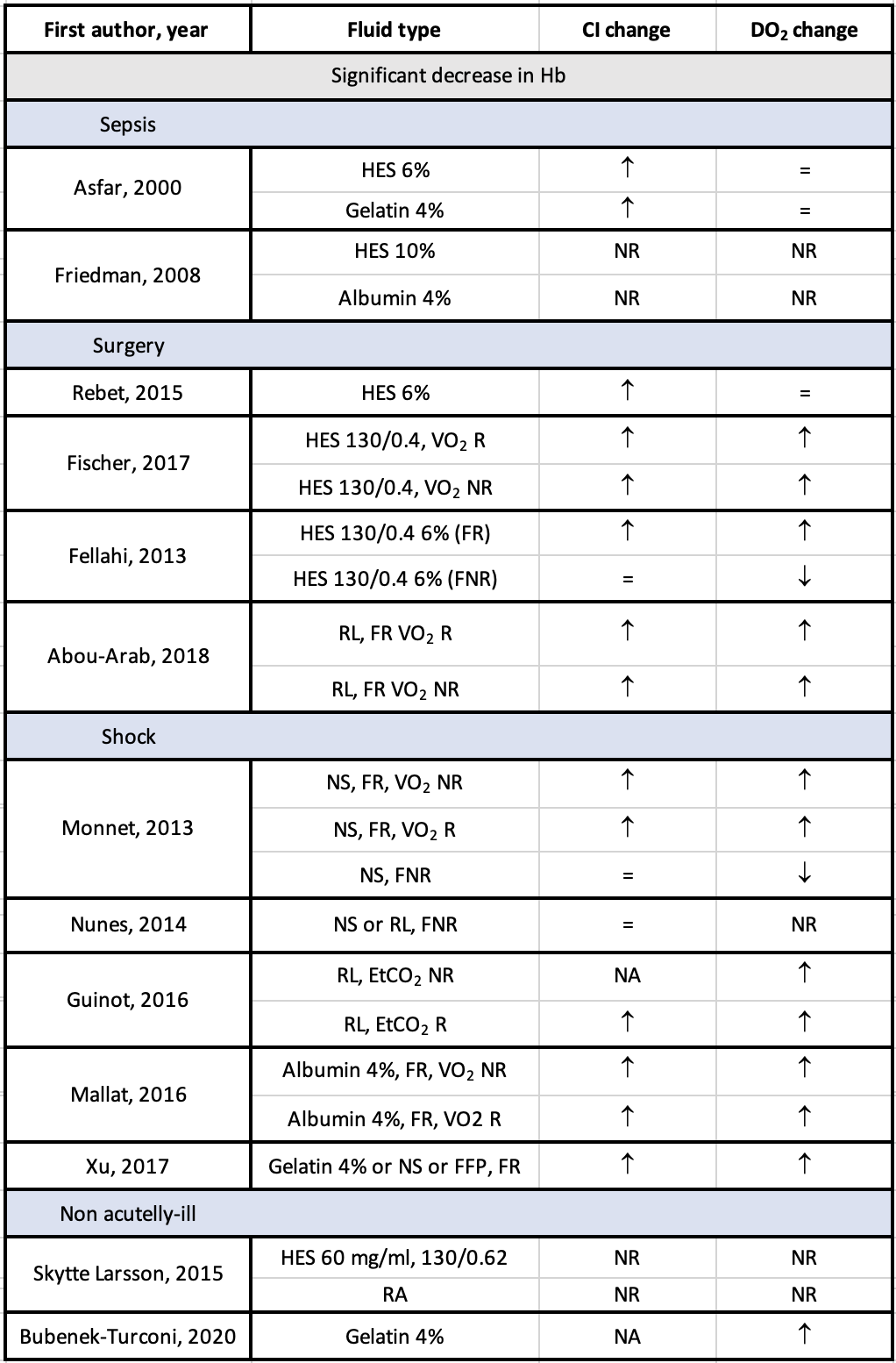

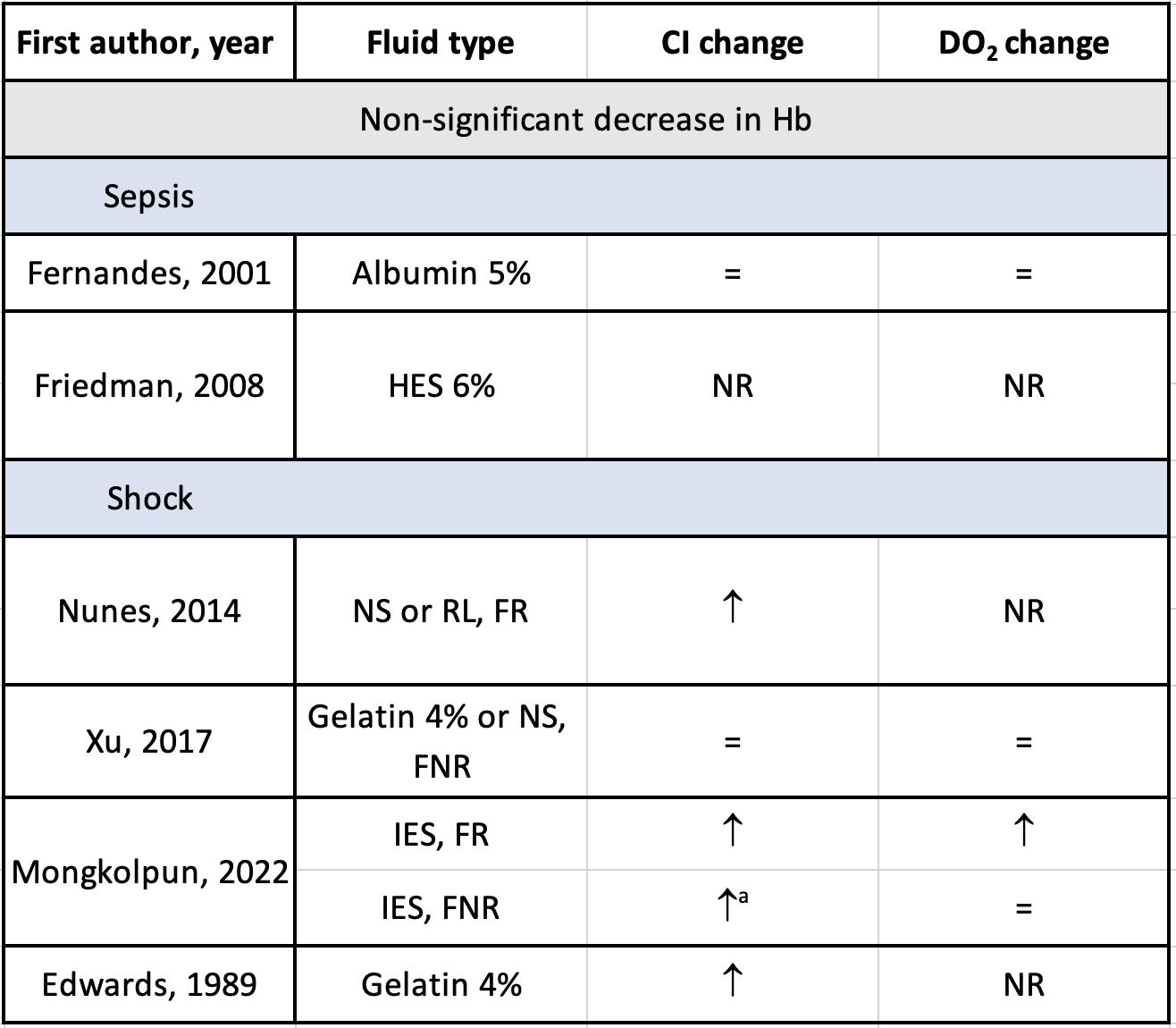


↑indicates increase; = indicates no change; ↓ indicates decrease; NA: Not applicable (fluid responders and fluid non-responders were mixed); NR: not reported; IES: isotonic electrolyte solution; NS: normal saline; RL: Ringer’s lactate; HES: hydroxyethyl starch; FFP: fresh frozen plasma; RA: Ringer’s acetate; FR: fluid responder; FNR: fluid non-responder; VO_2_R: oxygen consumption responders; VO_2_NR: oxygen consumption non-responders; EtCO2 NR: end-tidal carbon dioxide tension non-responders; EtCO2 R: end-tidal carbon dioxide tension responders. Positive and negative changes are reported according to p<0.05. ^a^Statistically significant increase but less than 15%.

# PRISMA checklist

| **Section and Topic** | **Item #** | **Checklist item** | **Location where item is reported** |
| --- | --- | --- | --- |
| **TITLE** | | |  |
| Title | 1 | Identify the report as a systematic review. | Page 1 |
| **ABSTRACT** | | |  |
| Abstract | 2 | See the PRISMA 2020 for Abstracts checklist. | Page 2 |
| **INTRODUCTION** | | |  |
| Rationale | 3 | Describe the rationale for the review in the context of existing knowledge. | Page 3 |
| Objectives | 4 | Provide an explicit statement of the objective(s) or question(s) the review addresses. | Page 3 |
| **METHODS** | | |  |
| Eligibility criteria | 5 | Specify the inclusion and exclusion criteria for the review and how studies were grouped for the syntheses. | Page 3 and Additional file 1 |
| Information sources | 6 | Specify all databases, registers, websites, organisations, reference lists and other sources searched or consulted to identify studies. Specify the date when each source was last searched or consulted. | Page 3 |
| Search strategy | 7 | Present the full search strategies for all databases, registers and websites, including any filters and limits used. | Page 3 and Additional file 1 |
| Selection process | 8 | Specify the methods used to decide whether a study met the inclusion criteria of the review, including how many reviewers screened each record and each report retrieved, whether they worked independently, and if applicable, details of automation tools used in the process. | Page 3 and Additional file 1 |
| Data collection process | 9 | Specify the methods used to collect data from reports, including how many reviewers collected data from each report, whether they worked independently, any processes for obtaining or confirming data from study investigators, and if applicable, details of automation tools used in the process. | Page 3 and 4 and Additional file 1 |
| Data items | 10a | List and define all outcomes for which data were sought. Specify whether all results that were compatible with each outcome domain in each study were sought (e.g. for all measures, time points, analyses), and if not, the methods used to decide which results to collect. | Page 4 |
|  | 10b | List and define all other variables for which data were sought (e.g. participant and intervention characteristics, funding sources). Describe any assumptions made about any missing or unclear information. | Pages 4 |
| Study risk of bias assessment | 11 | Specify the methods used to assess risk of bias in the included studies, including details of the tool(s) used, how many reviewers assessed each study and whether they worked independently, and if applicable, details of automation tools used in the process. | Page 4 and Additional file 1 |
| Effect measures | 12 | Specify for each outcome the effect measure(s) (e.g. risk ratio, mean difference) used in the synthesis or presentation of results. | Page 4 |
| Synthesis methods | 13a | Describe the processes used to decide which studies were eligible for each synthesis (e.g. tabulating the study intervention characteristics and comparing against the planned groups for each synthesis (item #5)). | Page 4 and Additional file 1 |
|  | 13b | Describe any methods required to prepare the data for presentation or synthesis, such as handling of missing summary statistics, or data conversions. | Page 4 and Additional file 1 |
|  | 13c | Describe any methods used to tabulate or visually display results of individual studies and syntheses. | Page 4 |
|  | 13d | Describe any methods used to synthesize results and provide a rationale for the choice(s). If meta-analysis was performed, describe the model(s), method(s) to identify the presence and extent of statistical heterogeneity, and software package(s) used. | Page 4 |
|  | 13e | Describe any methods used to explore possible causes of heterogeneity among study results (e.g. subgroup analysis, meta-regression). | Page 4 |
|  | 13f | Describe any sensitivity analyses conducted to assess robustness of the synthesized results. | Page 4 |
| Reporting bias assessment | 14 | Describe any methods used to assess risk of bias due to missing results in a synthesis (arising from reporting biases). | NA |
| Certainty assessment | 15 | Describe any methods used to assess certainty (or confidence) in the body of evidence for an outcome. | Page 4 |
| **RESULTS** | | |  |
| Study selection | 16a | Describe the results of the search and selection process, from the number of records identified in the search to the number of studies included in the review, ideally using a flow diagram. | Page 5 and figure 1 |
|  | 16b | Cite studies that might appear to meet the inclusion criteria, but which were excluded, and explain why they were excluded. | Figure 1 |
| Study characteristics | 17 | Cite each included study and present its characteristics. | Page 5 and table E2 |
| Risk of bias in studies | 18 | Present assessments of risk of bias for each included study. | Table E1 |
| Results of individual studies | 19 | For all outcomes, present, for each study: (a) summary statistics for each group (where appropriate) and (b) an effect estimate and its precision (e.g. confidence/credible interval), ideally using structured tables or plots. | Tables E2 and E10 |
| Results of syntheses | 20a | For each synthesis, briefly summarise the characteristics and risk of bias among contributing studies. | Table E1 |
|  | 20b | Present results of all statistical syntheses conducted. If meta-analysis was done, present for each the summary estimate and its precision (e.g. confidence/credible interval) and measures of statistical heterogeneity. If comparing groups, describe the direction of the effect. | Additional file 1 and figures 2 and 3 |
|  | 20c | Present results of all investigations of possible causes of heterogeneity among study results. | Page 6 |
|  | 20d | Present results of all sensitivity analyses conducted to assess the robustness of the synthesized results. | Page 7 |
| Reporting biases | 21 | Present assessments of risk of bias due to missing results (arising from reporting biases) for each synthesis assessed. | Page 7 |
| Certainty of evidence | 22 | Present assessments of certainty (or confidence) in the body of evidence for each outcome assessed. | Page 6 |
| **DISCUSSION** | | |  |
| Discussion | 23a | Provide a general interpretation of the results in the context of other evidence. | Page 9 and 10 |
|  | 23b | Discuss any limitations of the evidence included in the review. | Page 11 |
|  | 23c | Discuss any limitations of the review processes used. | Page 11 |
|  | 23d | Discuss implications of the results for practice, policy, and future research. | Page 12 |
| **OTHER INFORMATION** | | |  |
| Registration and protocol | 24a | Provide registration information for the review, including register name and registration number, or state that the review was not registered. | Page 3 |
|  | 24b | Indicate where the review protocol can be accessed, or state that a protocol was not prepared. | Page 3 |
|  | 24c | Describe and explain any amendments to information provided at registration or in the protocol. | NA |
| Support | 25 | Describe sources of financial or non-financial support for the review, and the role of the funders or sponsors in the review. | Page 12 |
| Competing interests | 26 | Declare any competing interests of review authors. | Page 12 |
| Availability of data, code and other materials | 27 | Report which of the following are publicly available and where they can be found: template data collection forms; data extracted from included studies; data used for all analyses; analytic code; any other materials used in the review. | NA |

# References

1. Ehrly AM, Landgraf H. Influence of intravenous infusions of hydroxyethylstarch (HES) (MW 40,000 and 450,000) on the blood flow properties of healthy volunteers. Angiology 1985; 36:41-4.

2. Ehrly AM, Seebens H, Saeger-Lorenz K. [Effect of a 10% and 6% hydroxyethyl starch solution (molecular weight 200,000/0.62) in comparison with a 10% dextran solution (molecular weight 40,000) on flow properties of blood and tissue oxygen pressure in patients with intermittent claudication]. Infusionstherapie 1988; 15:181-7.

3. Murray AM, Morgan M, Whitwam JG. Crystalloid versus colloid for circulatory preload for epidural caesarean section. Anaesthesia 1989; 44:463-6.

4. Stamler KD. Effect of crystalloid infusion on hematocrit in nonbleeding patients, with applications to clinical traumatology. Ann Emerg Med 1989; 18:747-9.

5. van den Broek WG, Trouwborst A, Bakker WH. The effect of iso-oncotic plasma substitutes: gelatine, dextran 40 (50 g/l) and the effect of Ringer's lactate on the plasma volume in healthy subjects. Acta Anaesthesiol Belg 1989; 40:275-80.

6. Dewachter P, Laxenaire MC, Donner M, Kurtz M, Stoltz JF. [In vivo rheologic studies of plasma substitutes]. Ann Fr Anesth Reanim 1992; 11:516-25.

7. Yamauchi H, Fukuyama H, Ogawa M, Ouchi Y, Kimura J. Hemodilution improves cerebral hemodynamics in internal carotid artery occlusion. Stroke 1993; 24:1885-90.

8. Berg S, Engman A, Hesselvik JF, Laurent TC. Crystalloid infusion increases plasma hyaluronan. Crit Care Med 1994; 22:1563-7.

9. Jung F, Meier C, Koscielny J, Pindur G, Moll A, Schimetta W et al. Effects of a hypervolemic hemodilution with HES 100/0.5 10% in patients with PAOD stage II: Elimination kinetics and blood fluidity. Clinical Hemorheology and Microcirculation 1996; 16:631-43.

10. Treib J, Haass A, Pindur G, Grauer MT, Wenzel E, Schimrigk K. Decrease of fibronectin following repeated infusion of highly substituted hydroxyethyl starch. Infusionsther Transfusionsmed 1996; 23:71-5

11. Comes L, Mureşan A, Costin Z. Observations on isovolemic hemodilution in acute ischemic stroke. Rom J Intern Med 1996; 34:43-7.

12. Kass LE, Tien IY, Ushkow BS, Snyder HS. Prospective crossover study of the effect of phlebotomy and intravenous crystalloid on hematocrit. Acad Emerg Med 1997; 4:198-201.

13. Sefrin P, Rauch S, Zieglmeyer C. [Changes in blood coagulation in treatment with hydroxyethyl starch]. Anaesthesiol Reanim 1998; 23:149-56.

14. Ueyama H, He YL, Tanigami H, Mashimo T, Yoshiya I. Effects of crystalloid and colloid preload on blood volume in the parturient undergoing spinal anesthesia for elective Cesarean section. Anesthesiology 1999; 91:1571-6.

15. Waters JH, Bernstein CA. Dilutional acidosis following hetastarch or albumin in healthy volunteers. Anesthesiology 2000; 93:1184-7.

16. Levin JM, Frederick Bde B, Ross MH, Fox JF, von Rosenberg HL, Kaufman MJ et al. Influence of baseline hematocrit and hemodilution on BOLD fMRI activation. Magn Reson Imaging 2001; 19:1055-62.

17. Lobo DN, Stanga Z, Simpson JA, Anderson JA, Rowlands BJ, Allison SP. Dilution and redistribution effects of rapid 2-litre infusions of 0.9% (w/v) saline and 5% (w/v) dextrose on haematological parameters and serum biochemistry in normal subjects: a double-blind crossover study. Clin Sci (Lond) 2001; 101:173-9.

18. Rehm M, Haller M, Orth V, Kreimeier U, Jacob M, Dressel H et al. Changes in blood volume and hematocrit during acute preoperative volume loading with 5% albumin or 6% hetastarch solutions in patients before radical hysterectomy. Anesthesiology 2001; 95:849-56.

19. Berg S, Golster M, Lisander B. Albumin extravasation and tissue washout of hyaluronan after plasma volume expansion with crystalloid or hypooncotic colloid solutions. Acta Anaesthesiol Scand 2002; 46:166-72.

20. Molter GP, Soltész S, Larsen R, Baumann-Noss S, Biedler A, Silomon M. [Haemodynamic effects following preoperative hypervolemic haemodilution with hypertonic hyperoncotic colloid solutions in coronary artery bypass graft surgery]. Anaesthesist 2003; 52:905-18.

21. Reid F, Lobo DN, Williams RN, Rowlands BJ, Allison SP. (Ab)normal saline and physiological Hartmann's solution: a randomized double-blind crossover study. Clin Sci (Lond) 2003; 104:17-24.

22. Kuipers H, Brouwer T, Dubravcic-Simunjak S, Moran J, Mitchel D, Shobe J et al. Hemoglobin and hematocrit values after saline infusion and tourniquet. Int J Sports Med 2005; 26:405-8.

23. Lehmann G, Marx G, Förster H. Bioequivalence comparison between hydroxyethyl starch 130/0.42/6 : 1 and hydroxyethyl starch 130/0.4/9 : 1. Drugs RD 2007; 8:229-40.

24. Ruttmann TG, Montoya-Pelaez LF, James MF. The coagulation changes induced by rapid in vivo crystalloid infusion are attenuated when magnesium is kept at the upper limit of normal. Anesth Analg 2007; 104:1475-80.

25. Borup T, Hahn RG, Holte K, Ravn L, Kehlet H. Intra-operative colloid administration increases the clearance of a post-operative fluid load. Acta Anaesthesiol Scand 2009; 53:311-317.

26. Lobo DN, Stanga Z, Aloysius MM, Wicks C, Nunes QM, Ingram KL et al. Effect of volume loading with 1 liter intravenous infusions of 0.9% saline, 4% succinylated gelatine (Gelofusine) and 6% hydroxyethyl starch (Voluven) on blood volume and endocrine responses: a randomized, three-way crossover study in healthy volunteers. Crit Care Med 2010; 38:464-70.

27. Chowdhury AH, Cox EF, Francis ST, Lobo DN. A randomized, controlled, double-blind crossover study on the effects of 2-L infusions of 0.9% saline and plasma-lyte® 148 on renal blood flow velocity and renal cortical tissue perfusion in healthy volunteers. Ann Surg 2012; 256:18-24.

28. Yamakage M, Bepperling F, Wargenau M, Miyao H. Pharmacokinetics and safety of 6 % hydroxyethyl starch 130/0.4 in healthy male volunteers of Japanese ethnicity after single infusion of 500 ml solution. J Anesth 2012; 26:851-7.

29. Andrijauskas A, Ivaškevičius J, Porvaneckas N, Stankevičius E, Svensen CH, Uvarovas V et al. A mini volume loading test for indication of preoperative dehydration in surgical patients. Medicina (Kaunas) 2015; 51:81-91.

30. Bihari S, Wiersema UF, Schembri D, De Pasquale CG, Dixon DL, Prakash S et al. Bolus intravenous 0.9% saline, but not 4% albumin or 5% glucose, causes interstitial pulmonary edema in healthy subjects. J Appl Physiol (1985) 2015; 119:783-92.

31. Andrijauskas A, Svensen CH, Porvaneckas N, Šipylaitė J, Stankevičius E, Činčikas D et al. A mini volume loading test (mVLT) using 2.5-mLkg(-1) boluses of crystalloid for indication of perioperative changes in hydration status. Medicina (Kaunas) 2016; 52:354-65.

32. Li Y, Shan Y, Lin X. Effect of acute hypervolemic hemodilution of 6% hydroxyethyl starch 130/0.4 on the EC(50) of propofol at two clinical endpoints in patients. Exp Ther Med 2016; 11:110-16.

33. Lee JH, Choo YJ, Lee YH, Rhim JH, Lee SH, Choi BM et al. Population-based volume kinetics of Ringer's lactate solution in patients undergoing open gastrectomy. Acta Pharmacol Sin 2019; 40:710-16.

34. Zdolsek M, Hahn RG, Zdolsek JH. Recruitment of extravascular fluid by hyperoncotic albumin. Acta Anaesthesiol Scand 2018; 62:1255-60.

35. Bihari S, Wiersema UF, Perry R, Schembri D, Bouchier T, Dixon D et al. Efficacy and safety of 20% albumin fluid loading in healthy subjects: a comparison of four resuscitation fluids. J Appl Physiol (1985) 2019; 126:1646-60.

36. Bradley CR, Bragg DD, Cox EF, El-Sharkawy AM, Buchanan CE, Chowdhury AH et al. A randomized, controlled, double-blind crossover study on the effects of isoeffective and isovolumetric intravenous crystalloid and gelatin on blood volume, and renal and cardiac hemodynamics. Clin Nutr 2020; 39:2070-79.

37. Yi JM, Bang JY, Choi B, Cho C, Lee YH, Lee EK et al. Population-based volume kinetics of crystalloids and colloids in healthy volunteers. Sci Rep 2019; 9:18638.

38. Bubenek-Turconi Ş I, Văleanu L, Popescu M, Panaitescu E, Tomescu D, Cacoveanu MC et al. Continuous noninvasive hemoglobin monitoring reflects the development of acute hemodilution after consecutive fluid challenges. Anesth Analg 2020; 130:696-703.

39. Hahn RG, Nemme J. Volume kinetic analysis of fluid retention after induction of general anesthesia. BMC Anesthesiol 2020; 20:95.

40. Li H, Bersten A, Wiersema U, Schembri D, Cavallaro E, Dixon DL et al. Bolus intravenous 0.9% saline leads to interstitial permeability pulmonary edema in healthy volunteers. Eur J Appl Physiol 2021; 121:3409-19.

41. Omar MN, Shouk TA, Khaleq MA. Activity of blood coagulation and fibrinolysis during and after hydroxyethyl starch (HES) colloidal volume replacement. Clin Biochem 1999; 32:269-74.

42. Awad S, Dharmavaram S, Wearn CS, Dube MG, Lobo DN. Effects of an intraoperative infusion of 4% succinylated gelatine (Gelofusine(R)) and 6% hydroxyethyl starch (Voluven(R)) on blood volume. Br J Anaesth 2012; 109:168-76.

43. Fellahi JL, Fischer MO, Rebet O, Dalbera A, Massetti M, Gérard JL et al. Cerebral and somatic near-infrared spectroscopy measurements during fluid challenge in cardiac surgery patients: a descriptive pilot study. J Cardiothorac Vasc Anesth 2013; 27:266-72.

44. Paydar S, Bazrafkan H, Golestani N, Roozbeh J, Akrami A, Moradi AM. Effects of Intravenous Fluid Therapy on Clinical and Biochemical Parameters of Trauma Patients. Emerg (Tehran) 2014; 2:90-5.

45. Rebet O, Fischer MO, Zamparini G, Gérard JL, Fellahi JL, Hanouz JL. Near-infrared spectroscopy hemoglobin index measurement during fluid challenge: a prospective study in cardiac surgery patients. J Cardiothorac Vasc Anesth 2015; 29:924-9.

46. Skytte Larsson J, Bragadottir G, Krumbholz V, Redfors B, Sellgren J, Ricksten SE. Effects of acute plasma volume expansion on renal perfusion, filtration, and oxygenation after cardiac surgery: a randomized study on crystalloid vs colloid. Br J Anaesth 2015; 115:736-42.

47. Fischer MO, Bonnet V, Lorne E, Lefrant JY, Rebet O, Courteille B et al. Assessment of macro- and micro-oxygenation parameters during fractional fluid infusion: A pilot study. J Crit Care 2017; 40:91-98.

48. Abou-Arab O, Braik R, Huette P, Bouhemad B, Lorne E, Guinot PG. The ratios of central venous to arterial carbon dioxide content and tension to arteriovenous oxygen content are not associated with overall anaerobic metabolism in postoperative cardiac surgery patients. PLoS One 2018; 13:e0205950.

49. Edwards JD, Nightingale P, Wilkins RG, Faragher EB. Hemodynamic and oxygen transport response to modified fluid gelatin in critically ill patients. Crit Care Med 1989; 17:996-8.

50. Bernard S, Buist M, Monteiro O, Smith K. Induced hypothermia using large volume, ice-cold intravenous fluid in comatose survivors of out-of-hospital cardiac arrest: a preliminary report. Resuscitation 2003; 56:9-13.

51. Meyer P, Pernet P, Hejblum G, Baudel JL, Maury E, Offenstadt G et al. Haemodilution induced by hydroxyethyl starches 130/0.4 is similar in septic and non-septic patients. Acta Anaesthesiol Scand 2008; 52:229-35.

52. Spiel AO, Kliegel A, Janata A, Uray T, Mayr FB, Laggner AN et al. Hemostasis in cardiac arrest patients treated with mild hypothermia initiated by cold fluids. Resuscitation 2009; 80:762-5.

53. Monnet X, Julien F, Ait-Hamou N, Lequoy M, Gosset C, Jozwiak M et al. Lactate and venoarterial carbon dioxide difference/arterial-venous oxygen difference ratio, but not central venous oxygen saturation, predict increase in oxygen consumption in fluid responders. Crit Care Med 2013; 41:1412-20.

54. Nunes TS, Ladeira RT, Bafi AT, de Azevedo LC, Machado FR, Freitas FG. Duration of hemodynamic effects of crystalloids in patients with circulatory shock after initial resuscitation. Ann Intensive Care 2014; 4:25.

55. Guinot PG, Guilbart M, Hchikat AH, Trujillo M, Huette P, Bar S et al. Association between end-tidal carbon dioxide pressure and cardiac output during fluid expansion in operative patients depend on the change of oxygen extraction. Medicine (Baltimore) 2016; 95:e3287.

56. Mallat J, Lemyze M, Meddour M, Pepy F, Gasan G, Barrailler S et al. Ratios of central venous-to-arterial carbon dioxide content or tension to arteriovenous oxygen content are better markers of global anaerobic metabolism than lactate in septic shock patients. Ann Intensive Care 2016; 6:10.

57. Xu B, Yang X, Wang C, Jiang W, Weng L, Hu X et al. Changes of central venous oxygen saturation define fluid responsiveness in patients with septic shock: A prospective observational study. J Crit Care 2017; 38:13-19.

58. Mongkolpun W, Gardette M, Orbegozo D, Vincent JL, Creteur J. An increase in skin blood flow induced by fluid challenge is associated with an increase in oxygen consumption in patients with circulatory shock. J Crit Care 2022; 69:153984.

59. Asfar P, Kerkeni N, Labadie F, Gouëllo JP, Brenet O, Alquier P. Assessment of hemodynamic and gastric mucosal acidosis with modified fluid versus 6% hydroxyethyl starch: a prospective, randomized study. Intensive Care Med 2000; 26:1282-7.

60. Fernandes CJ, Jr., Akamine N, De Marco FV, De Souza JA, Lagudis S, Knobel E. Red blood cell transfusion does not increase oxygen consumption in critically ill septic patients. Crit Care 2001; 5:362-7.

61. Matejovic M, Krouzecky A, Rokyta R, Jr., Novak I. Fluid challenge in patients at risk for fluid loading-induced pulmonary edema. Acta Anaesthesiol Scand 2004; 48:69-73.

62. Friedman G, Jankowski S, Shahla M, Gomez J, Vincent JL. Hemodynamic effects of 6% and 10% hydroxyethyl starch solutions versus 4% albumin solution in septic patients. J Clin Anesth 2008; 20:528-33.

63. Smart L, Macdonald SPJ, Bosio E, Fatovich D, Neil C, Arendts G. Bolus therapy with 3% hypertonic saline or 0.9% saline in emergency department patients with suspected sepsis: A pilot randomised controlled trial. J Crit Care 2019; 52:33-39.

64. Robba C, Messina A, Battaglini D, Ball L, Brunetti I, Bassetti M et al. Early effects of passive leg-raising test, fluid challenge, and norepinephrine on cerebral autoregulation and oxygenation in COVID-19 critically ill patients. Front Neurol 2021; 12:674466. 10.3389/fneur.2021.674466

65. Spoelstra-de Man AM, Smorenberg A, Groeneveld AB. Different effects of fluid loading with saline, gelatine, hydroxyethyl starch or albumin solutions on acid-base status in the critically ill. PLoS One 2017; 12:e0174507.
